# Quantifying wheat spike morphology by high resolution 3D surface scanning

**DOI:** 10.1186/s13007-026-01550-5

**Published:** 2026-06-15

**Authors:** Latifa Greche, Nicolas Virlet, Malcolm J. Hawkesford

**Affiliations:** Crop Resource Use and Quality, Rothamsted Research, West Common, Harpenden, AL5 2JQ UK

**Keywords:** Wheat, Spike shape, Surface scanning, Yield prediction, Mesh reconstruction, Trait extraction

## Abstract

An understanding of spike shape will be of great benefit for improving wheat yields. Traditional manual measurements of spike traits are slow and prone to human error, preventing large-scale phenotyping. Employing imaging techniques will allow researchers to measure multiple morphometric parameters simultaneously. While 2D imaging provides a rapid screening method, 3D imaging offer a more comprehensive understanding of spike shape, revealing complex external structures. This study addresses the challenge of developing a high-resolution 3D surface-scanning pipeline to accurately quantify wheat spike morphology across diverse genotypes. Using a 3D surface-scanner, sharp point clouds of individual spikes were reconstructed and automatically aligned and analysed to extract key morphological features including spike length, volume, and cross-sectional area profile. New shape descriptors based on cross-sectional area profiles, local extremes, statistical curve fitting, segmentation of spikes into zones of aborted spikelets, base and apical segments as well as the extraction of spike/spikelets branching and endpoints of components were introduced to capture detailed structural variation between genotypes. Correlations between the 3D-derived traits and traditional metrics such as spike weight, spikelet number and seed weight confirmed the biological relevance of the extracted parameters. The method distinguished morphological differences among twelve wheat genotypes, revealing distinct shape types such as long, short, compact, and awned spikes. By combining precise 3D imaging with computational analysis, this approach provides a non-destructive framework for spike phenotyping. These findings demonstrate that 3D surface-scanning can deliver accurate and reproducible measurements of wheat spike architecture, offering new opportunities for linking morphology with genetics and yield potential in modern breeding programs.

## Introduction

Wheat is a globally important cereal crop that plays a key role in food security. Enhancing crop productivity remains a central challenge in agricultural science. Among the various key traits influencing yield, spike morphology has emerged as a focal point for both researchers and breeders due to its direct link with important yield components such as grain number and grain weight. Morphological traits including spikelet number per spike (SNS), spike length (SL), spike density (SD), spike compactness (SCN), and awn presence [1] are tightly associated with yield potential, making them strategic targets in modern wheat breeding programs.

Numerous studies have explored the genetic basis of spike morphological traits across diverse wheat populations and environments. Through quantitative trait loci (QTL) mapping and genome-wide association studies (GWAS), researchers have identified multiple loci that control traits such as SL, SCN, and SNS, guiding breeding strategies that focus on enhancing grain yield through spike architecture optimization [2]. Traditional methods for quantifying spike morphology rely on manual measurement of spike length, width, and spikelet and kernel counts. While effective, these approaches are labour-intensive, time-consuming, and unsuitable for large-scale phenotyping. The increasing need for high-throughput analysis has driven the development of image-based phenotyping techniques that automate trait extraction.

Recent advances in computer vision using RGB imaging have enabled the automated assessment of traits such as spikelet number per spike through both in-field imaging systems [3–5] and controlled-environment setups [6, 7]. Additional efforts have focused on estimating traits such as grain number [8], grain size [9], and glume-based spike classification [10]. Using both side and front images of spikes [7] further enables the estimation of spike volume by partitioning the images into small cross-sections and computing the product of width, thickness, and length within each section. The Quadrangle model [11] exemplifies tools developed to extract a set of two-dimensional (2D) morphological traits from RGB images, including spike area, length, width, perimeter, roundness, circularity, and awn presence, while also classifying spikes into different shape types such as spelt, normal, and compact. This model has been applied to large genetic panels [12], contributing to the identification of genomic regions associated with both morphological and colorimetric spike traits.

However, 2D imaging lacks the capacity to fully capture the complex 3D structure of wheat spikes. Features such as 3D curvature, surface variation, and spatial thickness along the rachis are either underrepresented or completely missed. As a result, 3D imaging is increasingly recognized as a necessary evolution in spike phenotyping. Techniques such as X-ray computed tomography (CT) offer detailed internal and external views [13, 14], but suffer from high data volumes, low throughput, and intensive computational demands. A more scalable solution lies in 3D point cloud generation, typically via laser scanning or photogrammetry, which captures the external spike structure efficiently and yet with manageable data loads.

Recent research has leveraged 3D imaging for tasks such as spike segmentation [15, 16], spike counting [17], and morphological analysis [18–20]. Spike volume has been estimated using two main strategies: fitting multiple cuboids to the spike point cloud via the RANSAC algorithm and summing their volumes [18], or constructing a convex hull from triangulated surface points to approximate the volume [20]. More recently, spike volume has also been estimated using a hybrid deep learning approach [21] that combines DINOv2 [22] and Long Short-Term Memory (LSTM) networks [23]. This model was trained with multi-view RGB images of spikes paired with their corresponding 3D structured-light scans (surface point clouds), enabling accurate volume estimation. For spike length, Principal Component Analysis (PCA) is often applied to determine the spike’s primary axis, with length defined as the Euclidean distance between the base and tip along that axis [24]. When spikes are curved, a two-dimensional spline is fitted on the first and second PCA planes to estimate curved length [20]; however, this method ignores curvature in the third dimension, potentially underestimating true length. Width is typically extracted as the maximum distance across projections on the secondary or tertiary PCA components, but this approach fails to capture how width varies along the spike.

Most existing 3D phenotyping systems rely on static sets of 3D sensors [20, 25], which struggle to capture fine details of glumes, branching on the rachis, and awns from different views in dense plots. This is further complicated by occlusion and incomplete scanning of spikes in shorter tillers, as only spiles of taller tillers are fully exposed and able to be captured. Such limitations bias the phenotypic data, as longer tillers are known to support larger spikes with more spikelets [26, 27], likely due to superior light exposure and resource allocation. As the crop matures, spike curvature becomes more pronounced which complicate the morphology analysis [20]. To overcome these challenges, this study introduces a high-resolution 3D phenotyping pipeline designed for individual spike analysis across diverse genotypes. We focus on capturing complete and sharp detailed spike surfaces, overcoming occlusion and high plant density limitations, and enabling precise trait extraction. Specifically, we aim to:

- Utilize high-resolution 3D surface scanners (with resolutions up to 0.05 mm) to generate detailed point clouds of wheat spikes.
- Study a diverse wheat germplasm panel, including 11 Watkins landraces and the cultivar, Paragon, which together represent a broad spectrum of spike architectures such as short, awned, long, normal, and compact forms.
- Extract a comprehensive set of morphological traits, including spike volume, length, and cross-sectional area profile along the rachis.
- Statistically model cross-sectional area variation to derive new, informative morphometric descriptors.
- Perform trait correlation analysis to identify potential relationships between shape descriptors and yield-related metrics.

Integrating high-resolution scanning with detailed mesh-based morphometry, provides a robust framework for accurate spike analysis, contributing to improved phenotyping pipelines and enhanced genetic understanding of wheat spike architecture.

## Materials and Methods

### Materials

The field trials were conducted at Rothamsted Research, UK (51°48$'$34.56$''$N, 0°21$'$22.68$''$W), using the wheat cultivar Paragon and eleven genotypes selected from the Watkins landrace collection (Table 1; Fig. S1). The genotypes were sown in October 2021 and chosen to represent a wide range of spike morphologies. At physiological maturity, spikes were harvested from plots measuring 60 cm $\times$ 60 cm. For each genotype, approximately seven spikes were randomly sampled within the same plot to ensure representative data. The scanning process captured high-resolution 3D meshes for each spike, forming a robust dataset for subsequent analyses. The trial was conducted under rainfed conditions, and plants received standard agronomic management, including applications of sulphur (27 kg ha$^{-1}$), nitrogen (50 kg ha$^{-1}$), and appropriate autumn and spring herbicides, fungicides, and insecticides. Further information about the Watkins genotypes, including germplasm resources unit (GRU) store codes listed in Table 1, can be found in the SeedStor database [28].

**Table 1:** GRU store codes of the wheat cultivar Paragon and selected Watkins genotypes used for spike shape analysis

	WATDE0323	WATDE0930	WATDE0296	WATDE0228	WATDE0227	Paragon	WATDE0347	WATDE0748	WATDE0253	WATDE0045	WATDE0020	WATDE0354
Samples	8	7	8	9	9	8	9	5	7	8	7	7
Short	x						x					
Awned								x		x		

### Spike 3D surface data acquisition and reconstruction

**Fig. 1:**
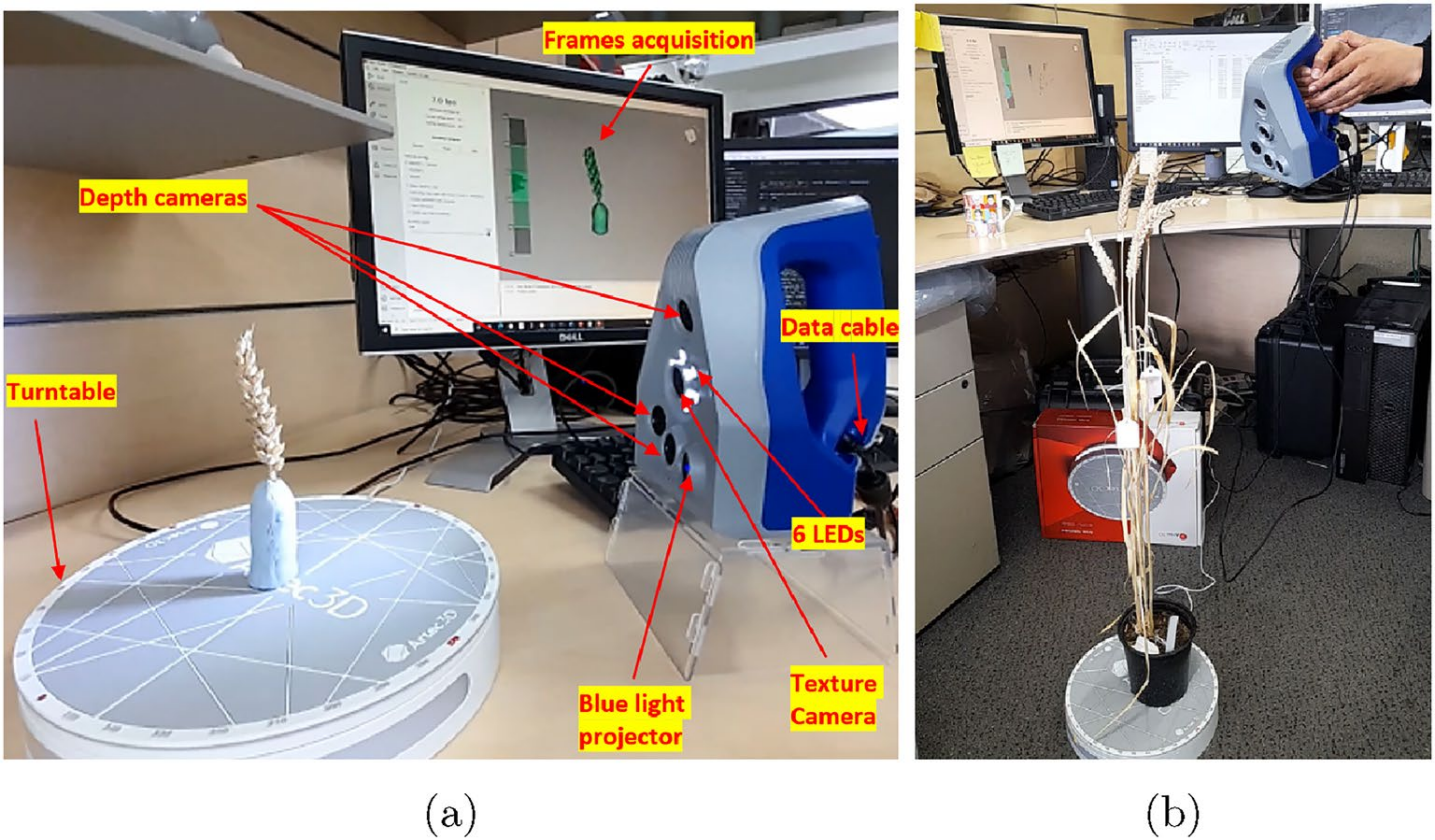
Data acquisition system: **a** Scaning an individual spike. **b** Scaning the whole plant

The experimental data was collected using a 3D surface scanner (Artec Space Spider, Artec 3D, Luxembourg, see Fig. 1), designed to capture high-resolution 3D scans, up to 0.1mm resolution, for small objects with complex fine details.

**Fig. 2:**
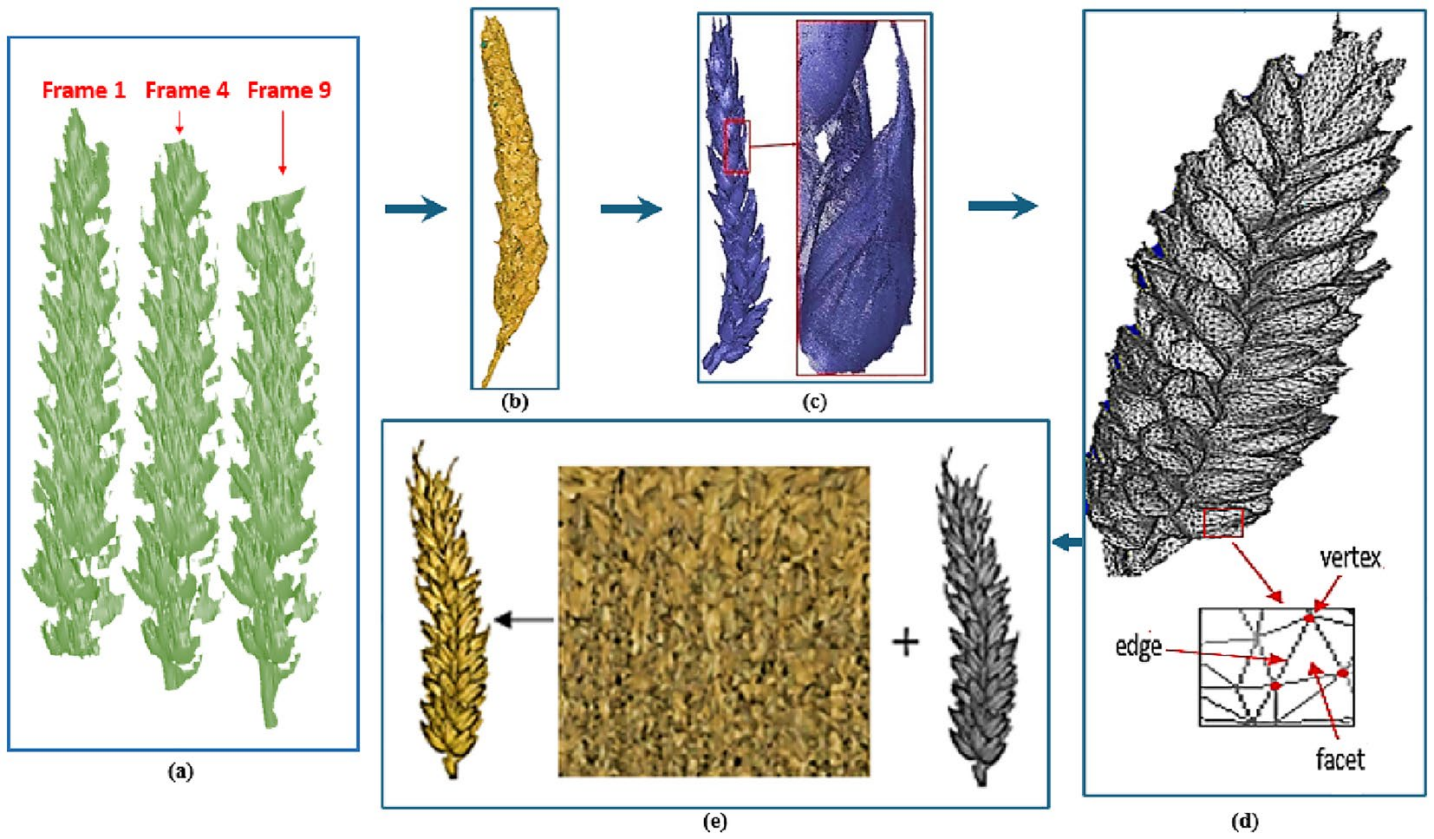
Overview of the preprocessing pipeline. **a** Example of frame surface recording. **b** Raw data after the completion the 3D surface scanning **c** A very dense 3D point cloud representation of the wheat spike after global registration and eliminating 3D noises. **d** Spike mesh after sharp fusion process for surface reconstruction. **e** Texture mapping to generate the mesh colour of the mature spike

The scanner consists of three depth cameras, an RGB camera (24 bpp with a resolution up to 1.3 MP), 6 LEDs and a blue light projector. During the surface scanning, one harvested spike is fixed in the centre of a turntable via a metal support. The turntable rotates with a constant speed and completes a full rotation after 60 s. While the spike is rotating around its central axis on the turntable, all the sensors in the scanner capture information simultaneously, and data is transferred as a single frame (Fig. 2a) to a computer linked to the handheld scanner via USB 3.0 (See data cable in Fig.  1a). Real time recorded frames are displayed (Artec Studio software, Artec 3D, Luxemburg) on the PC screen to enable the user to monitor the spike parts that have been scanned sucessfully.

At each frame, the scanner projects a grid pattern (reference pattern) of blue light. The benefit of the blue light is the shorter wavelength. Therefore, when the scanner projects light onto the spike, the shorter wavelength (of the blue light) allows it to diffract more sharply around small details. This finer diffraction enables the scanner to detect and record small variations in the spike surface geometry, capturing complex features with higher precision. Simultaneously, the three depth cameras observe the spike from three different angles and capture the reflected distorted light patterns. By measuring the time-of-flight of the reflected blue light as well as comparing the reference pattern with the distorted grid using triangulation, the 3D position of points on the spike surface and the distance of each point with the scanner are calculated.

**Table 2:** Average number of frames and points in spike for each variety

GRU code	AVG frame	AVG points (vertices)	Faces	Edges
WATDE0323	727.6	467346.8	934689.6	1402034.4
WATDE0930	553.1	96840.6	193677.2	290515.8
WATDE0296	649.3	117262.8	234521.6	351782.4
WATDE0228	657.3	98877.8	197751.6	296627.4
WATDE0227	696.7	143431.0	286858.0	430287.0
Paragon	396.8	118220.0	236436.0	354654.0
WATDE0347	671.4	199807.3	399610.6	599415.9
WATDE0748	732.4	169838.8	339673.6	509509.4
WATDE0253	732.0	120604.9	241205.8	361808.7
WATDE0045	864.4	178835.4	357666.8	536500.2
WATDE0020	598.0	104612.1	209220.2	313830.3
WATDE0354	436.4	42240.3	84476.6	126714.9

Depending upon the size of the spike (long, short, with/no awns or bent), the 3D scanner may capture several hundreds of frames (see Table 2) in three complete rotations of the turntable. For instance, for straight spikes without awns, the scanning can be stopped after 1 min, which is the time for the scanner to complete a full rotation while moving it up and down to enable recording all the spike details. However, the scanning of long, bent and awned spikes may take longer (2 to 5 min) to move the scanner into more view angles to scan the parts that are hidden by the awns and spike curvature. The Artec studio software stores the captured frames after calculating the 3D points coordinate at each frame. Using the software, users can then manually save the scanned raw data (Fig. 2(a)) in various file formats, such as “.obj”, “. ply”, “. stl”, so on. The software requires a laptop with high specification: processor of 12th Generation Intel$\circledR$ Core$^{TM}$ i9-12900HK (24MB Cache, up to 5.0 GHz, 14 cores); memory of 64 GB, 2 x 32 GB, DDR5, 4800 MHz, dual-channel; GPU of NVIDIA$\circledR$ GeForce RTX$^{TM}$ 3050 Ti, 4 GB GDDR6, 45 W. Once the scanning is finished, several data preprocessing steps are required to reconstruct the 3D shape. These are: a global registration of the frames, outlier removal, surface reconstruction and texture mapping. These steps require manual parameter testing and setting (see details in the following sections) and are achieved using the Artec studio software. Figure 2 illustrates the 3D spike representation after each preprocessing step.

#### Global registration

This step integrates all the raw data (Fig. 2b) into a unified coordinate system (Fig. 2c). This process relies on using information about the relative positions of each surface pair. The algorithm initiates this transformation by strategically selecting a set of particular geometry points within each frame. Subsequently, it applies a detailed search in an area of 1 mm (parameter chosen by the user) for matching pairs of points across two consecutive frames. This is because when the spike is rotating and the scanner captures multiple frames, each two consecutive frames will have common geometry points. Therefore, through the exact coordination of geometry points and the identification of mutual matches, the global-registration algorithm achieves a holistic fusion of the spike frames.

#### Spike denoising

During the scanning procedure, it is usual to encounter noisy data points within the captured scene, manifesting as small, unconnected surfaces from the main surface structure. The presence of such outliers can impact the accuracy of the reconstruction and subsequently affect the efficacy of phenotype extraction process. To address this issue, the Artec Studio outlier removal technique is employed, utilizing an algorithm measuring for each surface point $p_i$ the mean distances $\bar{d}_i$ between that point and $k=50$ neighbouring points, as well as the standard deviation of these distances. Points exhibiting mean distances exceeding a threshold defined by $\bar{d}_i > \mu _d + \lambda \cdot \sigma _d$, (where $\mu _d$ and $\sigma _d$ are the global mean and standard deviation of all point-to-neighbour mean distances, computed over $k=50$ nearest neighbours) are classified as outliers and eliminated. The parameter $\lambda$ is the standard deviation multiplier. We used a 3D-noise level of 3 ($\lambda = 3$), the most conservative setting, to avoid removal of fine structural features such as awn tips, glume edges, and rachis branches (where the point cloud density is low as not easly visible to the surface scanner).

#### Mesh reconstruction: sharp fusion

After acquiring the complete 3D point cloud of the spike and removing outliers, a surface reconstruction algorithm in the Artec software is used to reconstruct the underlying geometry of the spike. This is achieved by connecting the points with each other, forming triangular facets (Fig. 2d) using a ball pivoting technique. In this technique, an initial seed point is chosen from the input point cloud of the spike, serving as the starting point for mesh construction. A ball with a user-defined radius is then centred at this seed point, representing the region where potential pivot points can be found. The ball is rotated in 3D space to identify neighbouring points within its radius (1 mm set manually in Artec Studio software), and a pivot point is selected based on specific criteria, including forming a valid triangle with the seed point and another point in the mesh. This process iterates, with each new pivot point becoming the seed for the next iteration, gradually expanding the mesh by forming triangular facets between connected points. As the algorithm progresses, it constructs a mesh representation of the spike surface.

#### Texture mapping

The last pre-processing step is applied to recover the spike colour. The RGB camera of the scanner with the help of the 6 LEDs (Fig.  2a) that guaranty good illumination, capture the colour of the spikes. Initially, during the 3D scanning process, RGB colour information is captured simultaneously with the depth data, producing a point cloud where each point has associated colour values. Texture mapping assigns colour from the RGB images to the 3D mesh surface through UV unwrapping. For each mesh vertex $\textbf{v}_i = (x_i, y_i, z_i)$, a UV coordinate pair $(u_i, v_i) \in [0,1]^2$ (see Fig. 2e) is computed by projecting $\textbf{v}_i$ onto the closest registered RGB frame using the camera’s intrinsic matrix $\textbf{K}$ and the extrinsic transformation $[\textbf{R}|\textbf{t}]$ associated with that frame:

$$\begin{pmatrix} u_i \\ v_i \\ 1 \end{pmatrix} = \frac{1}{z_c} \textbf{K} \left( \textbf{R} \textbf{v}_i + \textbf{t} \right) , \quad z_c = [\textbf{R}\textbf{v}_i + \textbf{t}]_z$$  

The resulting $(u_i, v_i)$ values index into a 2D texture image that stores the aggregated RGB pixel values captured across all frames. Each mesh facet is then rendered by interpolating the UV coordinates of its three vertices and sampling the texture image bilinearly at those coordinates. In cases where a vertex is visible in multiple frames, the RGB values are blended using a weighted average, with weights proportional to the cosine of the viewing angle to reduce perspective distortion artefacts.

### Pipeline for extracting spike 3D morphometric traits

**Fig. 3:**
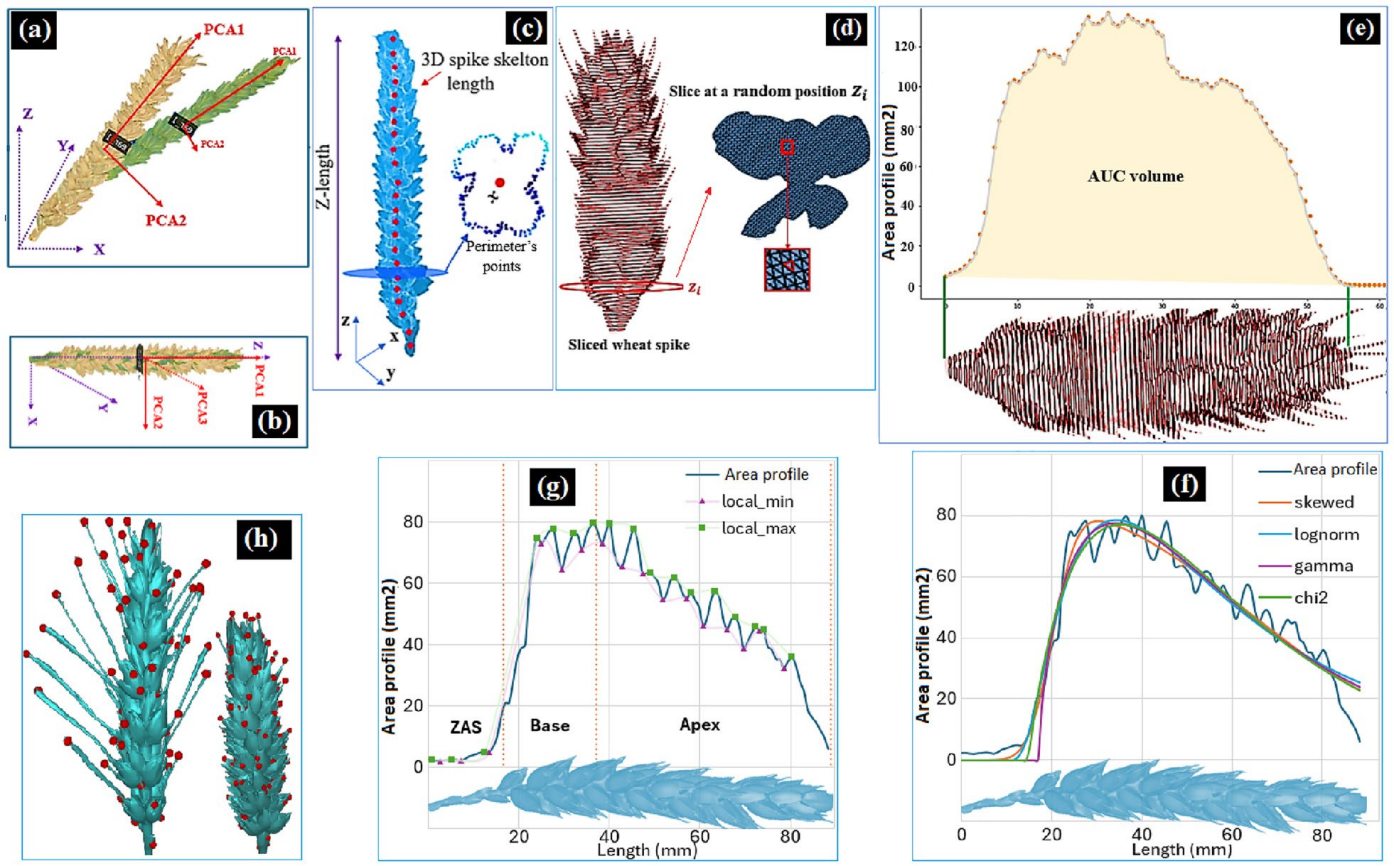
Pipeline overview for individual spike morphometric trait extraction. **a** Determination of the spike’s main axis. **b** Alignment of wheat spikes in the new coordinate system. **c** Length estimation using z-length and skeleton length. **d** Cross-sectioning of a short spike and measurement of slice areas (slice area). **e** Extraction of spike cross-sectional area distribution along a short spike and computation of volume. **f** Fitting of statistical models to spike area profiles and extracting statistical descriptors. **g** Detection of local extremes and segmentation of a long spike into the zone of aborted spikelets, base, and apex. **h** Extraction of connected branches and endpoint features of spike components (e.g. short dense and longawned spikes)

Figure 3 represents the main processing steps of the pipeline developed for extracting spike 3D morphometric traits from reconstructed wheat spikes. The process begins with defining the main axis of the spike allowing consistent orientation of all samples. Spikes are then aligned within a common coordinate system to ensure comparability across wheat genotypes. Following alignment, spike length is quantified using two methods (z-axis extent and the skeletonized structure) which are analysed and compared. Cross-sectioning is performed along the main axis (z-axis) to measure the area of individual slices, providing a detailed cross-sectional area profile along the spike. This profile curve is subsequently used to extract the spike volume and derive area characteristics. Multiple statistical models (skew-normal, lognormal, gamma, and chi-squared) are fitted to the cross-sectional area distribution of each spike to extract optimal parameters that capture tapering, shape and scale variability. Local extremes within the area profile are identified giving insights on spikelet density, and subsequently spikes were segmentated into three zones: zone of aborted spikelets (ZAS), base, and apex. Finally, connected skeleton branches and endpoint features corresponding to spike/spikelet components (rachis, glumes, lemmas, paleas, and awns) are extracted. Runing the pipline does not require a high specification computer. The pipeline was tested on a computer without GPUs with a processor of Intel$\circledR$ Core$^{TM}$ i7-7700 CPU @ 3.60 GHz (4 cores, 8 threads) and 16 GB of RAM.

#### 3D spike alignment

After completing the scanning, the orientation of the spikes is random in the 3D space. Aligning the wheat spikes according to a single reference orientation (*x*, *y*, *z*) helps normalization, as well as quantifying and comparing phenotypes. The alignment process begins by inferring the spike’s main axis of highest variance, then applying geometric rotations.

The principal component analysis (PCA) considers the spike as point cloud $P=\{(x_i, y_i, z_i) \mid i \in n\}$, where $(x_i, y_i, z_i)$ are point coordinates and *n* the number of points in the spike. Each point represents a data sample which helps finding the principal spike orientations sequentially one after another. The PCA looks at the point cloud from all possible angles to evaluate which of the infinite number of projections best seperates the data. This is done by resolving the generalised eigenvectors of the spike covariance matrix $\text {cov}(P)$ in $\text {cov}(P)\vec {V} = \lambda \vec {V}$ and selecting the eigenvectors, $\vec {V}$, that have the maximal eigenvalues, $\lambda$. The first three maximal eigenvalues $\lambda _1, \lambda _2, \lambda _3$ calculated represent the variance along each principal component axis $\vec {v}_1$, $\vec {v}_2$, and $\vec {v}_3$ respectively, as shown in image bellow. A larger eigenvalue corresponds to a higher variance which indicate the spike’s elongation over the new axis $\vec {v}_1$ (rachis axis, see Fig. 3a). By assuming the orthogonality of the three principal components $\vec {v}_1$, $\vec {v}_2$, and $\vec {v}_3$, this ensures that they are uncorrelated and each new axis (principal component) captures a different aspect of variation in the point cloud of the spike. To align $\vec {v}_1$ with the Z-axis, two angles $\alpha$ and $\beta$ are calculated as follow:$\alpha = \tan ^{-1}\left( \frac{v_{1y}}{v_{1x}}\right)$ and $\beta = \tan ^{-1}\left( \frac{\sqrt{v_{1x}^2 + v_{1y}^2}}{v_{1z}}\right)$.Where $\alpha$ is the angle between the projection of $\vec {v}_1$ on the XY-plane and $\beta$ is the X-axis and the inclination of $\vec {v}_1$ with respect to the Z-axis. The two angles are then employed to build a 3D rotation about the Y-axis (equation 4) and another 3D rotation about the Z-Axis (equation 5).

1 $$\begin{aligned} & R_y (\pi - \beta ) = \begin{bmatrix} \cos (\pi - \beta ) & 0 & \sin (\pi - \beta ) \\ 0 & 1 & 0 \\ -\sin (\pi - \beta ) & 0 & \cos (\pi - \beta ) \end{bmatrix} \end{aligned}$$

2 $$\begin{aligned} & R_z (-\alpha ) = \begin{bmatrix} \cos (\alpha ) & -\sin (\alpha ) & 0 \\ \sin (\alpha ) & \cos (\alpha ) & 0 \\ 0 & 0 & 1 \end{bmatrix} \end{aligned}$$

Then, a composite rotation of the spike is applied to align $\vec {v}_1$ with Z-axis by multiplying the transposed spike by the two calculated rotation matrices $R_y$ and $R_z$, $P' = R_y \times R_z \times P^t$

After aligning the rachis axis ($\vec {v}_1$) with Z-axis, there still be a roll about the Z-axis (which has become the rachis axis) to orient the secondary axis and align $\vec {v}_2$, and $\vec {v}_3$ with Y and X axis, respectively. Therefore, a second PCA has been applied on the rotated spike cloud $P'$ to obtain the new second eigenvector $\vec {v}_2$, compute the angle$\alpha ' = \tan ^{-1}\left( \frac{v_{2y}}{v_{2x}}\right)$ and apply a final roll rotation on the rachis axis by multiplying the rotation matrix $R_z (-\alpha ')$ by the spike cloud $P'$. This sequence of PCA-guided rotations ensures that all spikes are aligned in a consistent 3D pose. Finally, each spike is translated so that its centroid lies at the origin, establishing a shared spatial reference for the entire dataset as shown in Fig. 3b.

#### Spike length measurement

The length is relatively easy to measure if the spike is straight by projecting the spike elongation on the z-axis after the alignment process, see Fig. 3c. However, when the spike is curved, extracting the skeleton is required to achieve length measurement. Therefore, after slicing the spike mesh with 50 equally spaced planes parallel to xy-plan, the algorithm provides a set of points that intersect with the parallel plans, bringing the issue into a 2D space. Using the perimeter points of the sliced area as shown in Fig. 3c, the centroid point can be calculated by averaging the x and y coordinates of all the points to get the x and y position of the centroid. The spike skeleton length is then obtained by linking the centroid of the slices along z-axis and summing up the Euclidean distance between the adjacent centroids in 3D space.

**Fig. 4:**
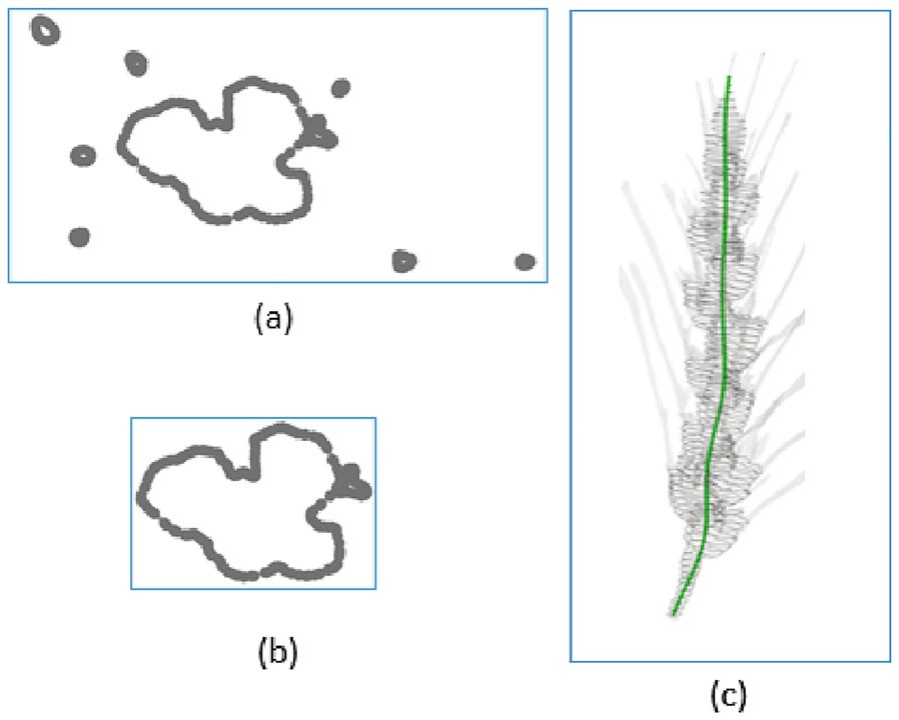
Example of skeleton length extraction for an awned spike: **a** Slice point cloud with awns. **b** Awns removed. **c** Spike and skeleton length visualization

For awned genotypes, awns intersect z-axis slicing planes, introducing small awn cross-sections into the slice (Fig. 4a) and displacing the centroid away from the rachis axis. To resolve this, each slice is first reduced to its largest component before centroid computation, allowing the elimination of small, disconnected components (awn cross-sections), as shown in (Fig. 4b). Then, centroid outliers, if still existing, are identified and removed using a median absolute deviation based modified z-score criterion [29] (threshold 3.5) applied independently to the x and y centroid sequences. The inlier centroids could also shift from the rachis axis to the center of spikelet slices if the distance between adjacent spikelets is higher (the case of low spike density). In this case, all the extracted inlier centroids are then smoothed using univariate splines fitted to x(z) and y(z) to produce the final centreline for length computation (Fig. 4c).

#### Spike cross-sectional area profiling

Spike cross-sectional area extraction requires applying a 3D cross-sectioning along the spike elongation axis (z-axis), in which the aligned spikes will be sliced into one hundred adjacent sections with planes parallel to xy-plane, see  3d. The spike slice area is then calculated as follow: for a 2D slice $S(z_i)$ at a given position $z_i$, $1 \le i \le 100$, the area is measured by splitting the slice surface into tiny triangles then summing up the area of all the triangle. $S(z_i) = \sum _{\text {all triangles}} \frac{b_{j,i} \cdot h_{j,i}}{2}$, where *b* and *h* are the triangle base and height, respectively. As each of the 2D slices can be represented by an area value, it is possible to extract and visualize the slice area over the hundred slices (Fig. 3e) to show the cross-sectional area distribution $y(z)$ measured along the spike’s longitudinal axis $z$ (base to apex).

**area profile descriptors**

Further analysis is caried out on the cross-sectional area distribution. For each spike, area profile was processed without smoothing or denoising. This is to keep the small curve fluctuation (as represented in Fig. 3(f)) that relates particularly to spikelet’s upper edges and the space between them, which is a good indicator of spikelet density in the spike. The identification of the curve fluctuations is done by the extraction of local extremes (minima and maxima), using a three-point rule: index $i$ is a local maximum if $y_i>y_{i-1}$ and $y_i>y_{i+1}$, and a local minimum if $y_i<y_{i-1}$ and $y_i<y_{i+1}$. Then counting the number of local extremes (NLE) which is the total of the number of the local minima and local maxima.

Using area profiles it was possible to split the spike into zone of abortive spikelets (ZAS), base, and apex see Fig. 3(f). This was done by locating and extracting the global curve peak $y_{\text {peak}}=\max _z y(z)$ and defining the ZAS–Apex boundary on projected z-axis of the spike as $z^{*} = \min \{\, z : y(z) > y_0 + 0.10\, (y_{\text {peak}}-y_0)\,\}$, where $y_0$ is the initial slice area value where z=0. The borders of the segments are defined as follow: $\text {ZAS}=[z_1,z^{*}]$, $\text {Base}=[z^{*}, z_{\text {peak}}]$, and $\text {Apex}=[z_{\text {peak}}, z_n]$. By using this borders, the extraction of segment descriptors was carried out. To automatically capture the variation in those segents, a set of morphological descriptors were extracted and summarised in Table 3.

**Table 3:** Segment-level features extracted from area profiles *y*(*z*) on ZAS, base, and apex

Descriptor	Mathematical formula	Description
Length (L)	$\Delta z = z_{\text {end}} - z_{\text {start}}$	Segment length along the spike z-axis
Start slice area (SSA)	$y_{\text {start}}$	slice area at segment start
End slice area (ESA)	$y_{\text {end}}$	Slice area at segment end
Mean slice area (SA)	$\bar{y} = \frac{1}{\Delta z} \int _{z_{\text {start}}}^{z_{\text {end}}} y(z)\,dz$	Average cross-sectional area over the segment
Slope (S)	$\frac{\Delta y}{\Delta z} = \frac{y_{\text {end}} - y_{\text {start}}}{\Delta z}$,$S<0$in Apex	Mean rate of thickening (base) or thinning (apex)
Area under the curve (AUC)	$\int _{z_{\text {start}}}^{z_{\text {end}}} y(z)\,dz$	Integrated cross-sectional area over the segment
Amplitude (A)	$y_{\text {end}} - y_{\text {start}}$,$A<0$in Apex	Net change in area profile
Relative change (RC)	$\frac{y_{\text {end}} - y_{\text {start}}}{y_{\text {start}}}$,$RC<0$in Apex	Change relative to start cross-sectional area
AUC-to-trapezoid ratio (AUCR)	$\rho = \frac{\text {AUC}}{0.5\, (y_{\text {start}} + y_{\text {end}})\,\Delta z}$	Shape index:$\rho \approx 1$linear,$\rho > 1$convex,$\rho < 1$concave
Variability (SD)	$\sigma (y) = \sqrt{\frac{1}{n}\sum _{i=1}^n (y_i - \bar{y})^2}$	Absolute within-segment variability
Variability (CV)	$\text {CV} = \frac{\sigma (y)}{\bar{y}}$	Relative variability (normalized by mean cross-sectional area)
Tapering index (TI, Apex only)	$\tau = \frac{y_{\text {start}} - y_{\text {end}}}{y_{\text {start}}}$	Relative narrowing during the thinning in Apex

**Statistical descriptors** When examining the area profiles along the spike axis, a consistent spatial patterns that appear to reflect systematic variation in spikelet size and arrangement is observed. However, the raw area profile often exhibited local fluctuations due to numerous small-scale extremes (see Fig. 3f), resulting in a noisy distribution. To better capture the underlying biological main shape and minimize the influence of local irregularities, the area profiles are modelled using continuous standard probability distributions. Specifically, we applied four statistical fitting functions: skewed Gaussian (SG), log-normal (logN), gamma, and chi-square (chi2) distributions, (see Table 4). This is to approximate the overall shape of each spike’s cross-sectional area curve along the spike.

**Table 4:** Representation of the statistical distributions used to model spike area profiles and the parameters estimated for each fit

Fitting model	Equation	Parameters to adjust according to spike shape
skewed Gaussian	$y(z) = A \, 2\,\phi \!\Big (\tfrac{z-\textrm{loc}}{\sigma }\Big )\,\Phi \!\Big (\alpha \tfrac{z-\textrm{loc}}{\sigma }\Big )$	A (Amplitude),$\alpha$(skew),$\mu$(loc),$\sigma$(scale)
logN	$y(z) = A \frac{1}{(z-\textrm{loc})\, s \sqrt{2\pi }} \exp \!\left( -\frac{(\ln (z-\textrm{loc}))^2}{2\,s^2}\right) , \; z>\textrm{loc}$	A, *s* (shape), loc, scale=$e^{\mu }$
gamma	$y(z) = A \frac{(z-\textrm{loc})^{k-1} e^{-(z-\textrm{loc})/\theta }}{\Gamma (k)\,\theta ^k}, \; z>\textrm{loc}$	A, *k* (shape), loc, scale=$\theta$
chi2	$y(z) = A \frac{(z-\textrm{loc})^{\nu /2 -1} e^{-(z-\textrm{loc})/2}}{2^{\nu /2}\Gamma (\nu /2)}, \; z>\textrm{loc}$	A,$\nu$(degree of freedom), loc

The fit curves will help smoothing (see Fig. 3f) the area profile and ignoring the local extremes variations that comes from spikelets edges (respresented in Fig. 3g). This process requires adjusting the models’ parameters iteratively to minimize the difference between the original area profile and the predicted values through Mean Squared Error (MSE) method. Once the distribution is fitted, its precision is evaluated by the coefficient of determination ($R^2$). Then the optimal parameters indicating different levels of skewness, peaks shift, mode, mean, scale and shapes in the area profile are used as statistical descriptors to reveal more morphology insights and quantifying the spike shape, see Table 5.

**Table 5:** Biological interpretation of the optimal fit parameters

Parameter	Mathematical role	Biological interpretation
*A*	Vertical scaling factor of the fitted distribution	Relates to the overall maximum cross-sectional area of the spike: Generally, larger *A* indicates thicker spikes
loc	Horizontal shift of the curve along *z*	indicates the position of the peak of thickening along spike length, reflecting how early or late maximum occurs
Scale	Controls the spread of the distribution	Reflects the effective length of the spike segment over which sectional area is spread: Large scale means gradual thickening/tapering; small scale means sharp transitions
Shape	Determines steepness and tail slope	controls the distribution of thickening vs tapering: high value means sharp peak; low value means flatter profile with longer tail
Skewness ($\alpha$)	Controls asymmetry of the distribution	Reflects spike asymmetry, whether the base-to-peak thickening is more gradual than the peak-to-apex tapering (positive $\alpha$ right-skewed profile and the tail is longer in the Apex, vice versa; $\alpha$ converging to 0 refers normal gaussian distribution which means symmetrical area profile)
degree of freedom (df)	Shape parameter in Chi2 distribution	Another indicator of both peak sharpness and tail behaviour, comparable to the Gamma shape parameter

#### Spike volume measurement

This metric involves, first, fitting the cross-sectional area scatter of the spike with a cubic spline function to generate a continuous curve as illustrated in Fig. 3e. Then, measuring the area under the cross-sectional area curve using the z-coordinates 0 and spike length value as parts of the interval for integrating the cubic spline ($v_{\text {spike}} = \int _0^{z_{\text {spike length}}} \text {spline}(S(z)) \, dz$). Another technique tested in python was the voxelization-based volume estimation. This technique consists of dividing the cubic bounding box of the spike into a grid of small alike voxels of a size equal to 0.5mm^3^. The voxels that include vertices (see Fig. 2d) along with the empty voxels inside the enclosed surfaces of the mesh receive a binary value of 1,while the remaining voxels inside the bonding box receive a binary value of 0. Volume is obtained by summing up the total number of voxels with positive value and multiplying it with voxel unit (0.5mm^3^). This provides a volume estimate based on the discrete spatial occupancy of the spike within the 3D voxel grid. This is a straightforward method, but accuracy depends on the voxel size; smaller voxels increase accuracy but also computational cost. The choice of 0.5 mm^3^ voxel size is a balance between accuracy and computational load. The volume of all wheat spikes was measured using three methods: AUC, voxelization, and Artec studio software. In addition to enabling the 3D scanning, Artec studio software can measure the volume of the spikes, but with no indication about which method is implemented in the software. Therefore, comparing the pair combination of the three ways of extracting the volume will help selection of a suitable method among the AUC-volume and the voxelization techniques.

#### Extracting branches and endpoints of the 3D spike components

As the visible spikelet components are the glumes, the lemmas, the paleas, and the awns, an algorithm has been developed to locate the tip of those components (called also endpoints). In addition to this, a more complex set of features were extracted that quantify the branching within the rachis, spikelets and awns. This process was realised by extracting a 3D centreline skeleton from rachis, spikelet components (the glumes, the lemmas, the paleas and the awns) mesh by using a topology-preserving thinning approach on a voxelized spike. First, the 3D point cloud of the spike is converted into a uniform voxel grid. Second, a standard 3D thinning algorithm is applied to peel off outer voxels layer by layer iteratively but keeps any voxel whose removal would change the spike’s topology (for example, it would break a branch or merge two parts). Thinning stops when only a one-voxel-thick feature remains that follows the center of the rachis and the branching with the spikelet components and awns when they are present. To make the process more stable on very thin areas, a light 3D closing beforehand to seal tiny pinholes is performed. Finally, the skeleton voxels are mapped back to real-world coordinates using the voxel grid’s transform and a simple graph with nodes is built. Nodes with three or more neighbours were labelled as branch points, and nodes with exactly one neighbour were labelled as endpoints as shown in Fig. 3h. The extraction of the connected skeleton of the rachis, the spikelet components and the awns with defined branches and tips, allowed calculation of the total number of branches (refered as Num-branch feature) and end points (refered as Num-endpoints feature) for each spike.

## Results

### Diversity using the extracted traits

**Fig. 5:**
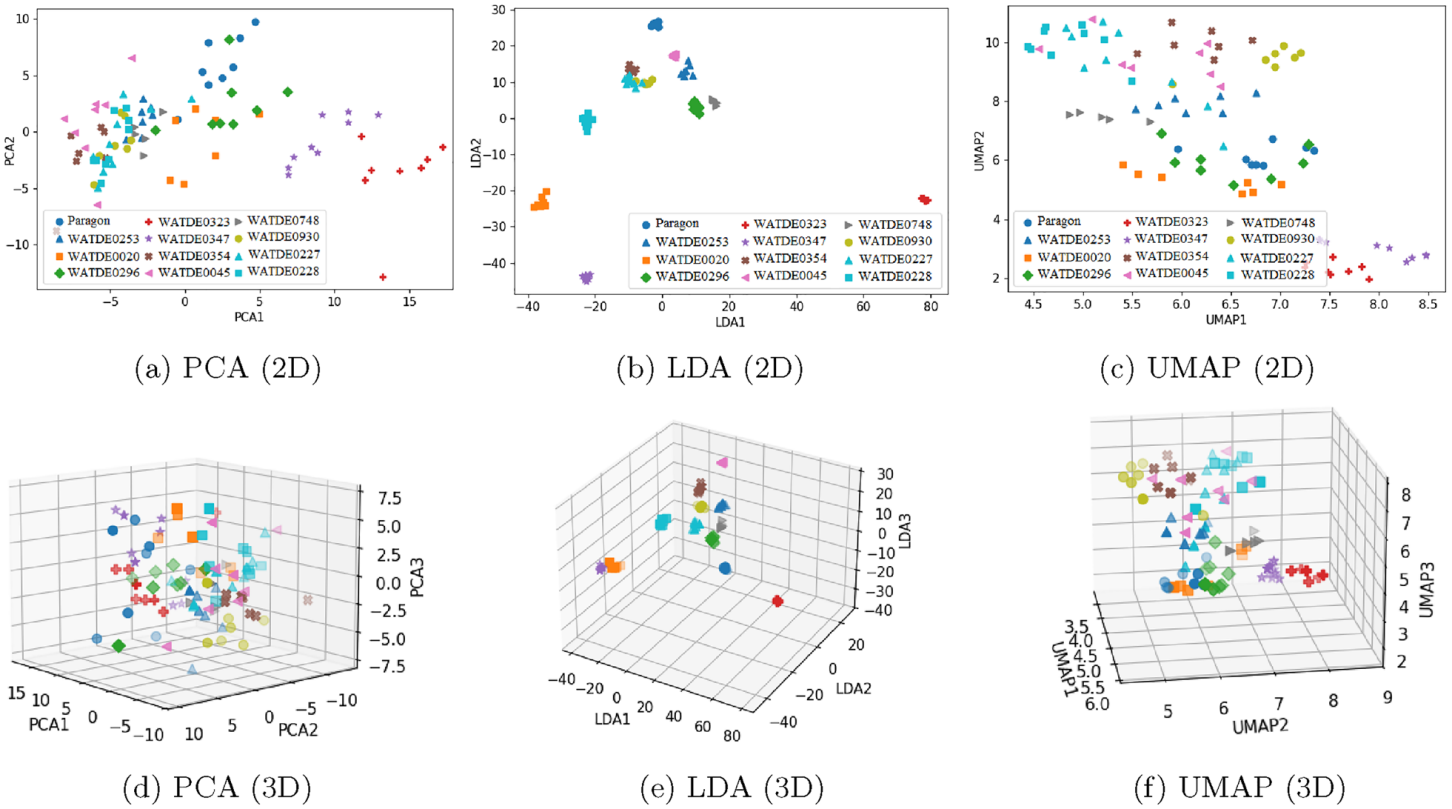
Embedding of all the extracted traits. Each plot displays the multidimensional relationships among spike morphological traits, reduced into two or three dimensions using: **a** 2D-PCA, **b** 2D-LDA, **c** 2D-UMAP, **d** 3D-PCA, **e** 3D-LDA, **f** 3D-UMAP. Points (spike samples) are coloured by genotype, demonstrating separability among varieties

The scatter diagrams in Fig. 5 demonstrate the diversity captured by all the extracted traits (see Table S6 in supplementary material) when projected into lower dimensions using linear unsupervised (PCA) and supervised (LDA) methods, as well an unsupervised non-linear (UMAP) method. The spread of points in PCA (Fig. 5aand d) shows that there is variation among genotypes (specifically, WATDE0323 and WATDE0347), but the overlap in most genotypes reflect the inability to discriminate these genotypes. In contrast, the LDA (Fig. 5band e) projections highlight that the dataset contains distinct sources of variation, with genotypes occupying more separated clusters particularly in the 3D LDA scatter. The spread in UMAP (Fig. 5cand f) provides an additional perspective by emphasizing local structures: some genotypes form compact clusters with internal homogeneity such as WATDE0323, WATDE0347, WATDE0748, and WATDE0930, others are more scattered, meaning there is higher within-variety variability.

### Comparing spike length methods

**Fig. 6:**
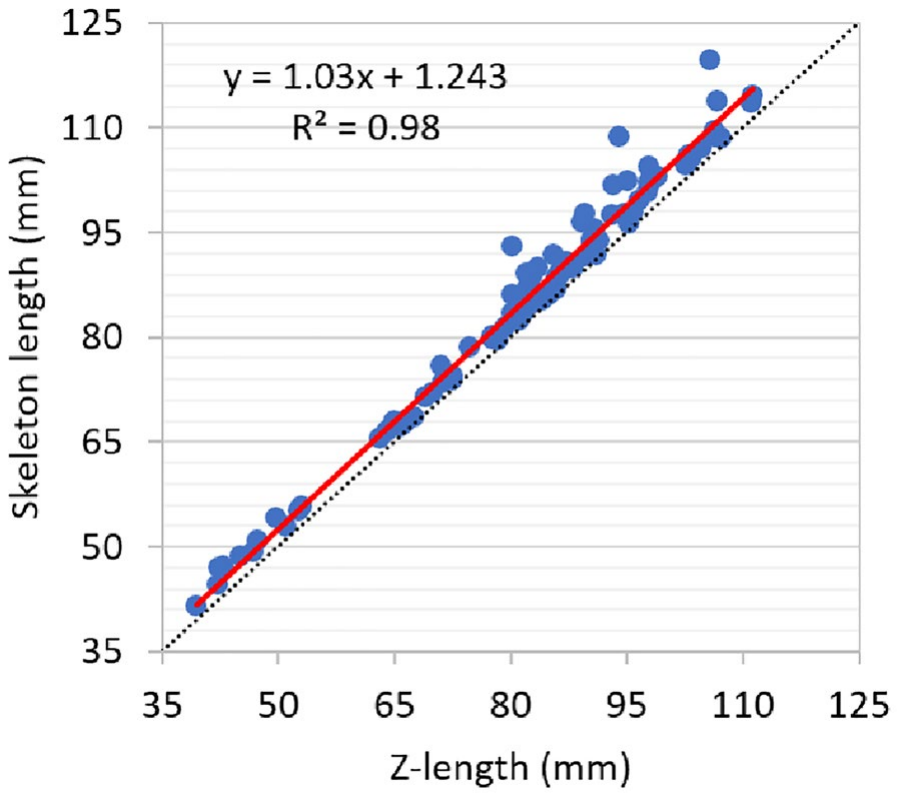
Correlation between skeleton length and the z-length of all the spikes

Figure 6 provides a comparison between two methods for measuring spike length: the traditional projected Z-length (vertical height of the spike) and the skeleton length, which is extracted from the internal structure of the spike. Both methods gave similar length measurements as demonstrated by the regression equation ($y=1.03x+1.24$) and the strong correlation R²$=$0.98. In table S7 (see supplementary material in excel file), across all spikes, projected z-length and skeleton length showed a mean bias of 3.45 mm, a mean absolute error (MAE) of 3.45 mm, and a root mean square error (RMSE) of 4.30 mm, indicating that the two computational length estimates were in close agreement in absolute terms. The length is slightly higher when using the skeleton approach which can better correct for curvature. A few outliers can be observed and correspond to spikes that are curved more than the others.

**Fig. 7:**
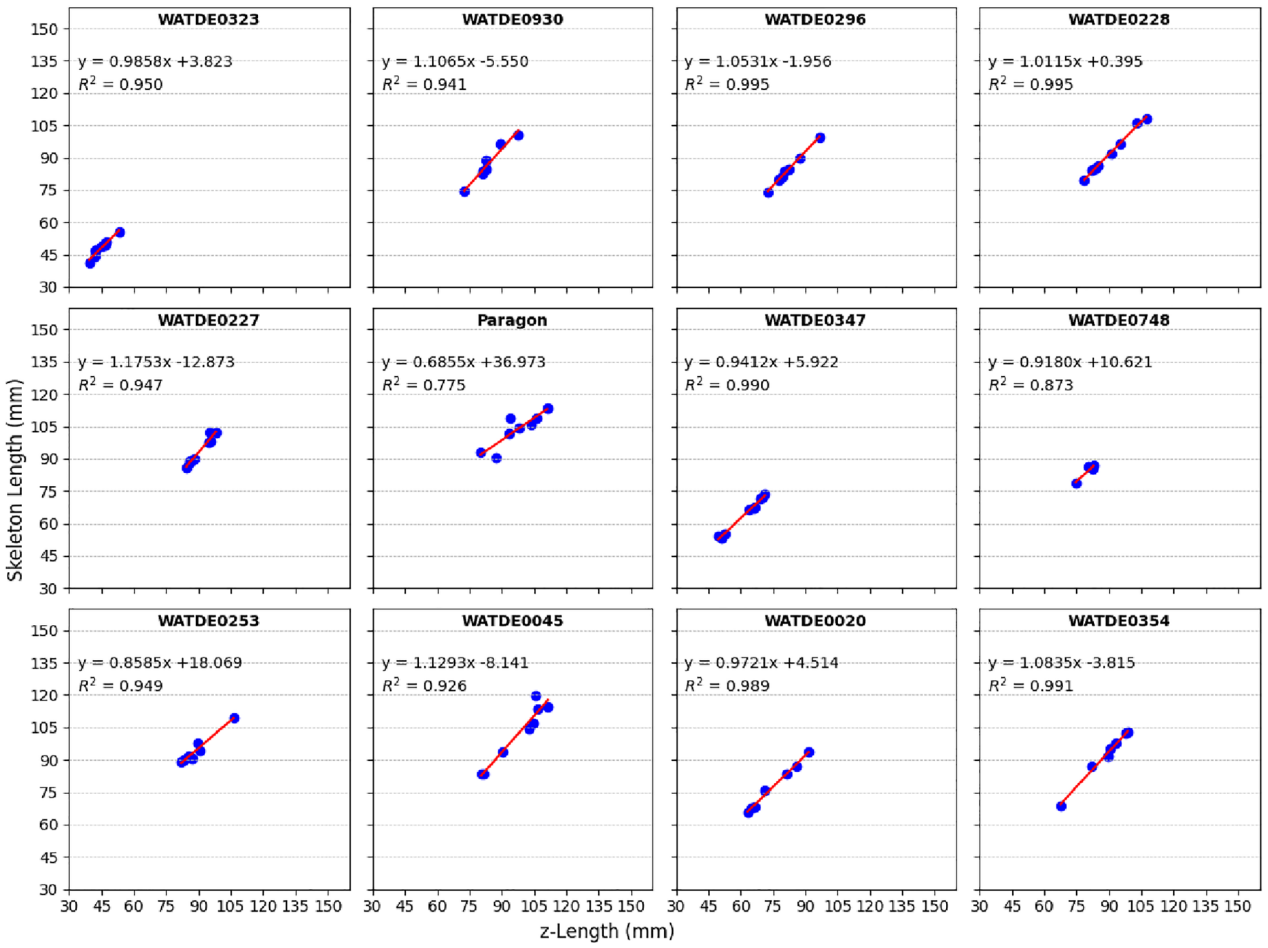
Relationship between the skeleton length and the projected length within genotypes

When the length is analysed for the individual genotypes (Fig. 7), the correlations remained high (R² > 0.7 for all cases), although slopes and intercepts varied slightly among the genotypes, suggesting small genotype-specific differences in how spike curvature contributes to skeleton and projected length measurements.

The genotypes show slopes varying from 0.68 to 1.17. Except for Paragon and WATDE0748, all the other genotypes show R²$\ge$0.92, reflecting consistency in spike length measurement. Genotypes, such as WATDE0228 (R² $=$ 0.99 and a $=$ 1.01), exhibit an almost perfect 1:1 correlation, indicating that the spikes in this variety are straight. Paragon (R²$=$0.775 and slope a$=$0.68) shows an average correlation, indicating that some spikes in this variety are curved or twisted, making projected length a less reliable measurement. In this case, the skeleton length captures the true spike length more precisely by accounting for the structure and curvature.

### Analysis of cross-sectional area descriptors

**Fig. 8:**
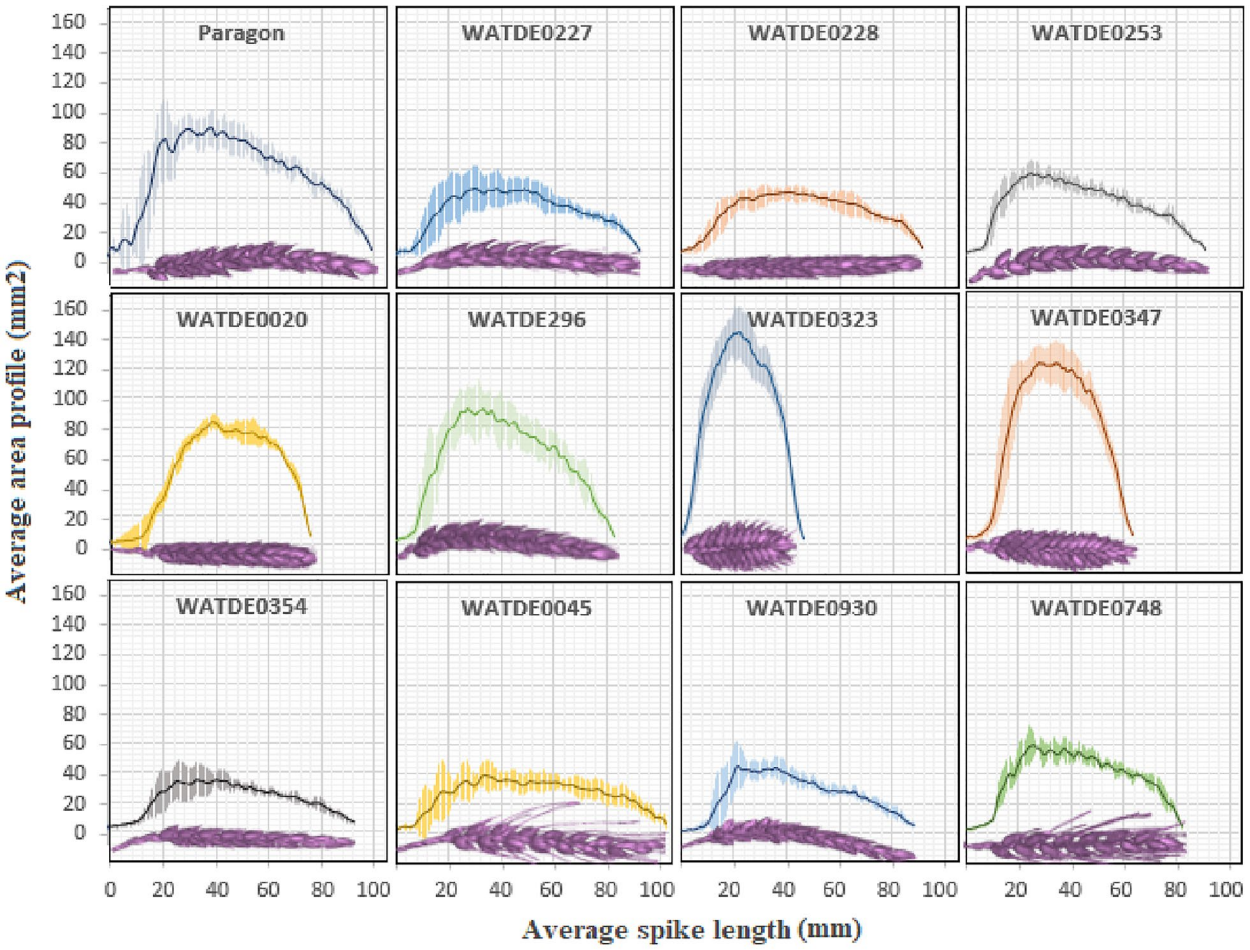
Each plot represents the mean cross-sectional area distribution along the average spike length for individual genotypes, including Paragon and Watkins lines. The shaded areas indicate the variability (± standard deviation) observed among the measured spikes of each genotype. Differences in curve shapes and peak heights reflect variations in spike morphology across genotypes shown by an example of 3D spike surface scan. Area profiles of individual spikes are in Fig. S3

The average area profiles in genotypes, in Fig. 8, revealed distinct morphology and shape differences, see Fig. S3 for individual spike cross-sectional area. In all cases, slice area increased from the base toward the midsection and decreased toward the apex, though the shape of the distribution varied. Some genotypes like WATDE0020, WATDE0354, WATDE0045, WATDE0930, and WATDE0748 have slightly longer lengths in the zone of aborted spikelets. The length profile of WATDE0323 and WATDE0347 have the appearance of a bell, with the highest peaks and shortest lengths, while the other genotypes have longer tails indicating thickening of the spikes between midsection and the top of the spike.

#### Local extremes in area profile and segment related traits

**Fig. 9:**
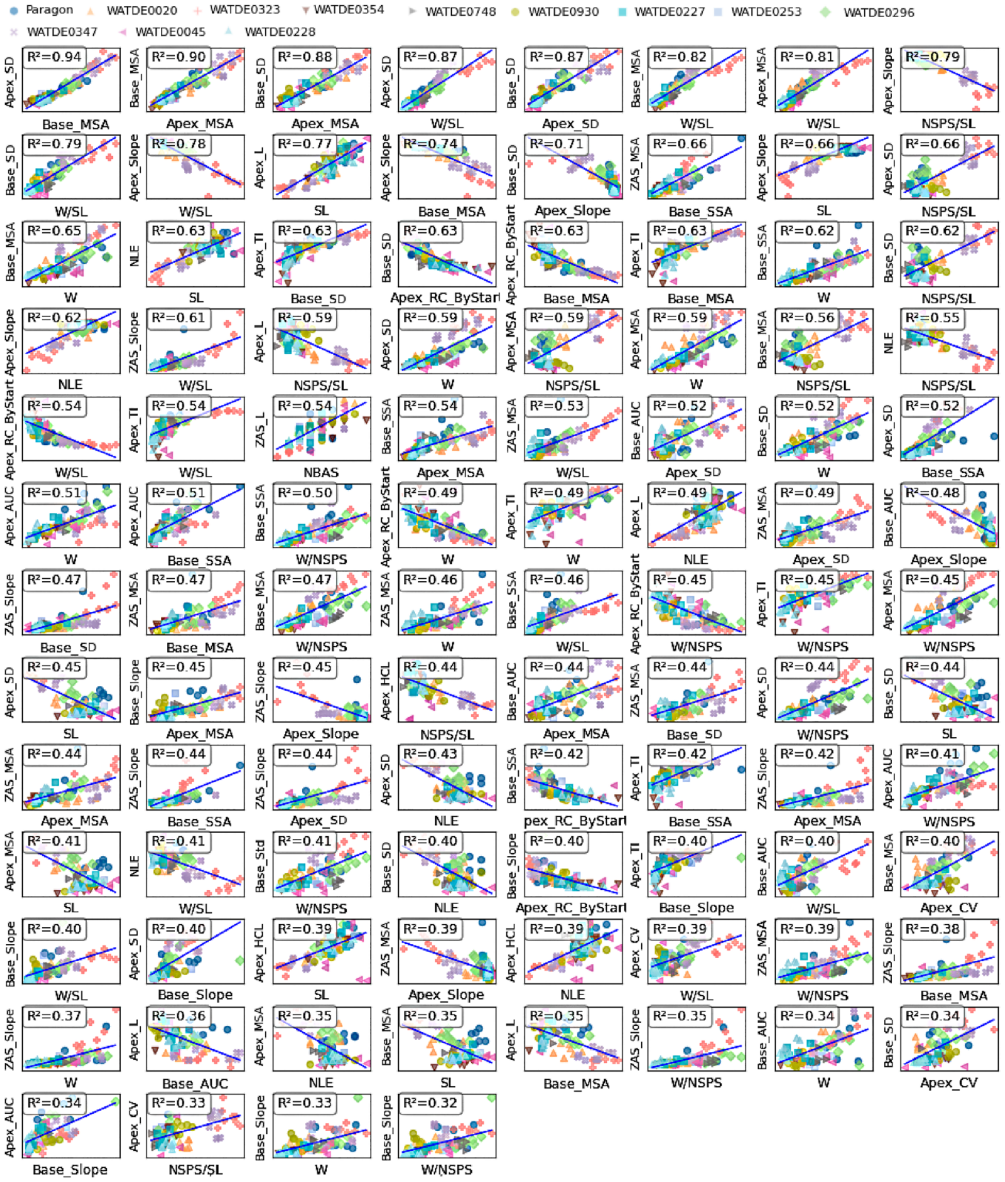
Pair scatter of segment-related traits, spike weight (W), number of spikelet per spike (NSPS), number of basal aborted spikelets (NBAS), and the number of local curve extremes (NLE). Each subplot represents the correlation between two traits, with fitted regression blue line and corresponding coefficients $R^2$ measured for all genotypes together. Different markers and colours correspond to individual genotypes, allowing visualisation and comparison of inter-genotypic variation

Figure 3g illustrates the segmentation of a spike into three structural regions: the ZAS, the base, and the apex, derived from area profiles. The ZAS is characterized by very low and stable slice area values, however the base and the apex have a more variable profile with multiple small fluctuations that are detected by the local extremes method. In Fig. 9 and Table S17, correlations within and across segments demonstrated that base mean slice area (Base-MSA) and base variability (Base-SD) are highly and positively correlated with apex counterparts ($R^2>0.87$). Base-MSA was also moderately associated with spike weight ($R^2=0.79$), spike skeleton length ($R^2=0.65$), and the ratio of weight to skeleton length (*W*/*SL*, $R^2=0.74$).

Apex mean slice area (Apex-MSA) was strongly correlated with weight ($R^2=0.78$) and W/SL ($R^2=0.71$). Apex slope (rate of tapering) correlated positively with NLE ($R^2=0.62$) and negatively with NSPS/SL ($R^2=0.79$), indicating that steep apical tapering is associated with lower spikelet density. The length of the zone of aborted spikelets (ZAS-L) correlated positively with the number of manually counted aborted spikelets in the base (NBAS, $R^2=0.54$), validating slice area-derived segmentation as a reliable predictor of spikelet abortion. ZAS-related ratios also showed moderate correlations with NSPS/SL and W/NSPS. NLE correlated positively with spike length (SL, $R^2=0.63$) and Apex-Slope ($R^2=0.62$), but negatively with spikelet density (NSPS/SL, $R^2=0.55$). These results suggest that spikes with more undulations in their area profile tend to be longer, with steeper apical tapering, but lower spikelet density. Thus, NLE (Extracted in Table S9) may serve as an indirect proxy for spikelet density. When analysing genotype-specific spread in the scatter plots of Fig. 9, most of the genotype spread evenly along the regression trendline. However, in some cases short dense genotype (WATDE0323 and WATDE0347) consistently clustered at the high values of NSPS/SL end of these correlations. This indicates a distinct pattern that can be useful for classification according to spike shape.

#### Fit-curve traits related area profile

**Fig. 10:**
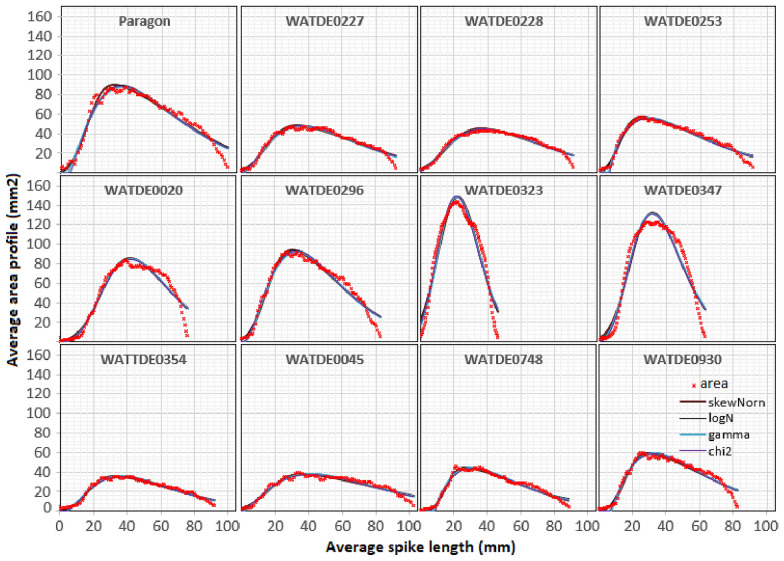
The plots illustrate the average spike area profile (red points) along the average spike length for each genotype, overlaid with the following statistical distribution models: skew-Gaussian, lognormal, gamma, and chi-square distribution. The fitted curves demonstrate how different probability distributions capture the asymmetry and variability of spike slice area along the spike long axis. These fits were used to evaluate model suitability for characterizing genotype-specific morphological patterns (see Fig. S3 of individual spikes)

To extract features using statistical distributions and quantitatively characterize the area profile of the spikes, each was fitted with the following statistical distributions: skewed Gaussian, log-normal, gamma, and chi2 models. For visualisation, Fig. 10 illustrate only the fits of the averaged cross-sectional area of each variety. Detailed information on the fits of individual spikes is provided in the supplementary materials for readers who are interested in the full data (see Table S10 and S15). For all genotypes, the fitted curves (Fig. 10 and Fig. S3) closely overlapped the average area profiles, with $R^2$ values above 0.8 (Tables  S1–S4 for averaged spike area profiles and S10 for individual spike area profile) for most genotypes, indicating strong model performance. The genotype WATDE0045 showed lower fitting performance (moderate $R^2$ values in Table S10) probably due to the high fluctuations observed in its area profile (see Fig. S3) rather than the limitation of the fitting-models in accurately capturing the spike’s area profile pattern within this genotype.

Although the fitted curves appeared visually similar across models, the optimal parameters extracted from the fits (Tables  S1–S4 and Table S10) could provide additional description of the spike morphology. For instance, the skewness parameter ($\alpha$) derived from the skewed-norm fit (Table S10) quantifies the degree of asymmetry in the profiles. Higher skewness values were observed in spikes from certain genotypes such as WATDE0045 (spike 5, $\alpha =71.44$ and $R^2=0.63$), WATDE0296 (spike 6, $\alpha =37.41$ and $R^2=0.86$), Paragon (spike 5, $\alpha =32.20$ and $R^2=0.87$), and WATDE0930 (spike 1, $\alpha =28.95$ and $R^2=0.91$). These high skewness values indicate more asymmetric area profile distributions, charaterized by higher slice area at the spike base compared to the apex, as shown in Fig. S3. Conversely, spikes within WATDE0323 genotyshow lower values of skewness ($\alpha$ convergoing to zero; see Table S10), and its corresponding skewed-normal with the Gaussian fits overlapped closely (Fig. S3), suggesting a symmetrical area profile from base to apex in this genotype. An exception was observed for WATDE0323 spike 2, which exhibited a negative skewness value, indicating greater slice area near the apex (Fig. S3).

Similarly, the scale parameters from all the four fits in Tables  S1–S4 and Table S10 reflected differences in spike relative length where spikelets are well developed. Genotypes with larger scale values (WATDE0045, WATDE0228, WATDE0227 and Paragon) shows broader central regions in Fig. 10. The amplitude is a scaling parameter of the area profile where it increases relatively with volume and weight (see, Fig. S5). In the chi2 fits (Tables S4 and S10), the degrees of freedom (df) varied between genotypes. A higher value in this parameter (WATDE0323, WATDE0347,and WATDE0020) indicates a symmetrical profile with sharp area curve peak and short length. while a smaller value reflects an asymmetrical profile with tapering in the apex of the spike.

Importantly, these parameter sets provide quantitative trait values that extend beyond simple curve visualization. For instance, in Table S10, most of genotypes displayed relatively higher skewness and scale values, reflecting elongated but asymmetric spikes, whereas WATDE0323 and WATDE0347 exhibited lower skewness and scale values, consistent with their more symmetrical compact and peaked profiles. Across all models, the relatively low mean square error (MSE) values confirmed that the fitting adequately captured variation in the observed area profiles.

### Comparing spike volume estimates

**Fig. 11:**
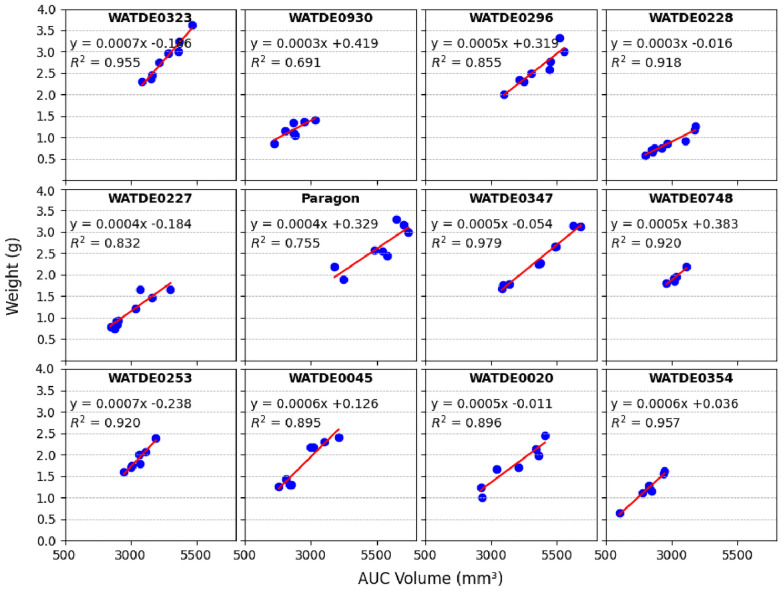
Scatter plots illustrating the relationship between spike weight and AUC-volume within each genotype

Spike volume was measured using three different methods: AUC integration, voxelization, and Artec-based measurement. All three approaches showed a near-perfect correlations (R² $\ge$ 0.99) and regression slopes close to 1 (Fig. S2). This confirms the robustness of the volumetric estimates and supports the comparability of different measurement techniques for capturing spike volume. When analysed individually per variety (Fig. 11), the correlation between weight and AUC-derived volume varied among genotypes.

In general, high coefficients of determination were observed ($R^2 = 0.69-0.98$) with the lowest for Paragon and WATDE0930 (0.75 and 0.69, respectively) and the highest for WATDE0347,176 and 202($R^2 = 0.98$, 0.92 and 0.92, respectively). The relationship shows that both weight and volume are increasing in parallel. The analysis of individual weight-to-volume relationships highlights key differences between the commercial variety Paragon and the Watkins landraces. Paragon shows a high range of spike weights varying from 1.88mg to 3.29mg (see Table S13) and moderate efficiency in weight increase ($slope = 0.41$). in addition, the short variety WATDE0323 has heavier spikes with a weight range varying from 2.3mg to 3.63mg and achieves the highest slope $a=0.7$ which indicates a potential for grain production as this variety increases weight rapidly with a minor increase in volume. WATDE0296 and WATDE0347 also exhibit a moderate efficiency in weight increase ($a = 0.481$ and $a= 0.50$ respectively), and a spike weight variation that is much closer to Paragon’s spike weight variation. For the remaining Watkins genotype, the spikes are generally lighter compared to Paragon. This suggests lower overall yield potential despite its consistency, as indicated by a $R^2$ value higher than 0.8 for most of this genotype. In terms of within-variety variation, Paragon shows more scatter, indicating that spike weights are less predictable. Conversely, WATDE0323 and WATDE0347 display much higher consistency ($R^2=0.96$).

### Analysis with spike branches and spikelet end points

**Fig. 12:**
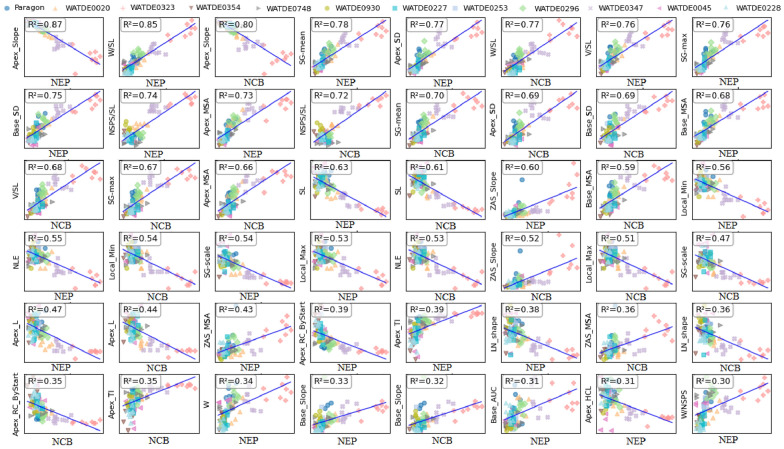
Correlation plots of spike branches and endpoint features (plots x-axis) with the previously extracted features (plots y-axis)

The features Num-branch and Num-endpoints (see Table S14) were correlated with all the previously extracted traits as well as the manually recorded traits (W and NSPS in Table S13). Figure 12 shows only the top correlations with an $$R^2>=0.30$$ between these two features and the rest of all the extracted traits. These two features highly correlated with each other ($$ R^{2}=0.96$$). Additionally they showed good correlations with the following features: Base-traits ($$0.68 \le R^{2} \le 0.77 $$), Apex-traits ($$0.73 \le R^{2} \le 0.88 $$), local extremes traits ($$0.53 \le R^{2} \le 0.57$$), the spike skeleton-length and its ratio with AUC-volume, W and NSPS ($$0.63 \le R^{2} \le 0.85 $$), and statistical descriptors ($$0.76 \le R^{2} \le 0.78 $$).

Moderate correlations of the Num-branch and the Num-endpoints ($R^{2}=0.52$–0.60) were observed with the ZAS-Slope, suggesting that branching complexity also scales with the degree of spike elongation and the longitudinal gradient of area profile along the spike. In contrast, the number of local extremes (NLE) showed a negative correlation with both branch number and endpoints ($R^{2}=0.54$–0.55), implying that spikes with smoother cross-sectional area profiles (i.e., fewer Local-Maxima) tend to have more organized branching and a higher number of end points.

## Discussion

### Morphological diversity of wheat spikes revealed by 3D traits and statistical models

Understanding the genetic impact on wheat spike shape requires analysis of the morphology of multiple genotypes. This process is best approached using 3D surface analysis. This study demonstrates the potential of high-resolution 3D scanning to extract a wide range of morphological traits from wheat spikes with high precision. By reconstructing 3D shape of wheat spikes and developing the tools, many sophisticated traits were extracted. These include, mainly, the number of connected branches of spikelets components and the rachis, the number of the end points representing the tip of glumes, lemmas, pales and awns in the spikelets, and the spike cross-sectional area profile. In addition, a set of cross-sectional area based traits was derived, including volume (AUC), local extremes, and spike segment traits such as slice area relative change, mean slice area, slope, tapering index, and within-segment variability (SD and CV) across the zone of aborted spikelets, basal, and apical regions. Further traits derived from cross-sectional area fitting curves were extracted (such as skewness, scale, peak location, mode, mean, degree of freedom) capturing information about whether the spikes have parallel-sided, pyramidal, long, short or compact shape.

The diversity analysis (with LDA, PCA and UMAP) shows the pattern and the complexity in the data while using all the extracted traits. The PCA scatter showed the maximum variance in the data regardless the spike variety. Although clustering for some genotypes was observed, the linearity in PCA is a major limitation where it led to miss detecting non-linear pattern in the data and overlaps with this technique appear mostly when groups differ in more complex way. The LDA model, by contrast, uses genotype labels to maximise the variance between the genotypes and minimise the within-variety variance. The resulting scatter plots showed the presence of meaningful diversity rather than random noise with the possibility of using the extracted traits for classifying (as the boundaries between spikes of each genotype are well defined) spike genotypes according to their shape traits. The non-linearity in the UMAP model help capturing more complex patterns in the data that PCA cannot. It shows diversity in terms of clusters and relative closeness, such which spikes/genotypes are close in trait space.

A potential limitation of this study is the relatively small number of spikes analysed per genotype. However, the focus of this work was to build the 3D phenotyping pipeline to help extract spike morphologies, rather than to perform definitive genotype analysis. Future work will deploy the pipeline to much larger datasets, where its usefulness for genetic analysis and breeding applications can be more fully assessed.

### Length phenotype

The strong correlations observed between skeleton length and projected length across all genotypes confirm that the 3D pipeline provides consistent structural descriptors of spike elongation. They reveal a strong positive correlation between these two measurements across all wheat genotypes. The skeletonization approach has important strengths: it accounts for 3D curvature of the rachis and is more accurate than projected z-length for bent or twisted spikes, as confirmed by the near-perfect R² = 0.98 correlation. However, it has known limitations. In genotypes having awns (e.g., WATDE0748, WATDE0045), z-axis slices intersect awn structures, displacing centroids from the rachis axis. The pipeline addresses this through per-slice small components filtering and median absolute deviation based centroid outlier removal prior to centreline smoothing using spline-fitting. Despite these improvements, very dense awn touching surface of spikelet could still partially bias the centreline estimate, and a dedicated awn masking step based on 3D segmentation of awn versus rachis components would provide a solution for future pipeline versions. The slight overestimation of skeleton length relative to z-length is consistent with the expected behaviour: skeleton length accounts for curvature that the projected z-length does not.

### Cross-sectional area phenotypes

The analysis of area profile and their fitted statistical models revealed variation not captured by length alone. Parameters such as skewness, spread, and area profile peak describe differences in compactness and asymmetry between genotypes, which may relate to differences in grain arrangement and density. These findings suggest that cross-sectional area related traits could serve as phenotypic markers for spike architecture in breeding programs.

An interesting observation from Fig. S5 and Table S16 is that base length shows no strong correlation with any other trait, suggesting that basal segment extension varies independently of other architectural or area-related characteristics. In contrast, Apex length is strongly positively correlated with skeleton length and the scale parameter of the fitting curves and negatively correlated with spikelet density (NSPS/SL).

Both base and apex mean slice area were strongly correlated with some fitting-curves parameters (mean, maximum, and amplitude) and ratios of volume and weight per length unit, as well as with spikelet density and spike weight, volume, number of endpoints, and number of branches. These relationships highlight that thicker spikes tend to be heavier, slightly denser and morphologically more complex.

Similar to base length, the Base-Slope trait showed weak correlations with other parameters, indicating that basal thickening dynamics are relatively independent. In contrast, the Apex-Slope displayed strong correlations with spikelet density, number of endpoints, number of branches, number of local extremes, skeleton length, and fit-curve parameters (mean and maximum). This suggests that apical slope is tightly linked to both spike structural complexity and the global shape of the cross-sectional area profile.

The association of the number of local extremes with spikelet density per length unit is particularly interesting: spikes with higher density exhibited fewer local extremes, suggesting that local extremes may be used as an indicator of spikelet density in the spike. The negative relationship between NLE and branching traits reveals that spikes exhibiting smoother cross-sectional area profiles (very smaller local oscillations) develop more structured and complete branching architectures.

### Volume

The volumetric analysis provides an integrative measure of spike size that is relevant, as indicated by the positive correlations with spike weight. The AUC-Volume approach has been used because it does not require access to the full 3D mesh to estimate spike volume. Unlike voxelization, which depends on storing and processing large 3D point clouds, the AUC-Volume method relies only on the area profile (a vector of 100 values per spike) making it computationally efficient and easy to apply across large datasets. Once the area profiles are extracted, volume can be computed directly from their area under the curve without the need for processing 3D surface scans.

### Future directions

The correlations observed between ZAS, base, and apex traits (Fig. S5, Table S16) indicate that spike slice area is globally coordinated along the spike axis. For example, the mean slices area within the ZAS increases proportionally with both base and apex, as indicated by fit-curve and segment-derived parameters such as amplitude, mean area, and maximum area. These relationships extend to whole spike features, where higher ZAS means cross-sectional area is associated with higher weight and volume per unit length. Spikelet abortion (caused by stress, resource competition, or genetic factors) has been widely studied in wheat. The approach proposed here provides a possible way to detect and quantify basal spikelet abortion through the ZAS length, which shows a correlation with the manually counted number of basal aborted spikelets (NBAS; Table S16). In some genotypes, aborted spikelets may also occur in the apex segment, which is an improvement that should be carried out in future versions of the proposed method. This would require improving the pipeline by adding a 3D spikelet segmentation task and extracting morphometric traits to help observing how those metrics change along the spike rachis.

Overall, the findings in this paper highlight the utility of 3D scanning for wheat spike phenotyping and demonstrate how computational tools can extract new morphological traits with potential relevance for yield prediction and genetic studies. Future work should focus on linking these traits to underlying genetic variation and agronomic performance, thereby integrating high-resolution phenotyping into breeding pipelines. In addition, applying the approach under field conditions or across developmental stages could further extend its applicability for capturing dynamic changes in spike growth and architecture.

## Additional file

Supplementary file 1 (xlsx 294 KB)

Supplementary file 2 (xlsx 885 KB)

Supplementary file 3 (xlsx 92 KB)

Supplementary file 4 (xlsx 14 KB)

Supplementary file 5 (pdf 2934 KB)

## Data Availability

Sample data are shared in the following link: https://github.com/LatifaGreche/3D-WheatSpikeMorphologyExtraction/tree/main/Data The codes are available at the following link: https://github.com/LatifaGreche/3D-WheatSpikeMorphologyExtraction

## Declarations

### Code Availability

The codes are available at the following link: https://github.com/LatifaGreche/3D-WheatSpikeMorphologyExtraction
