# Supplementary material for: Quantifying wheat spike morphology by high resolution 3D surface scanning
Source: Plant Methods. 2026 Jun 15;22:55. doi: 10.1186/s13007-026-01550-5 (PMC13267510; doi:10.1186/s13007-026-01550-5)
Supplement: Supplementary file 5 — (pdf 2934 KB) [file 13007_2026_1550_MOESM5_ESM.pdf]

1 Annex

Supplementary Materials

1.1 Diversity in wheat shape

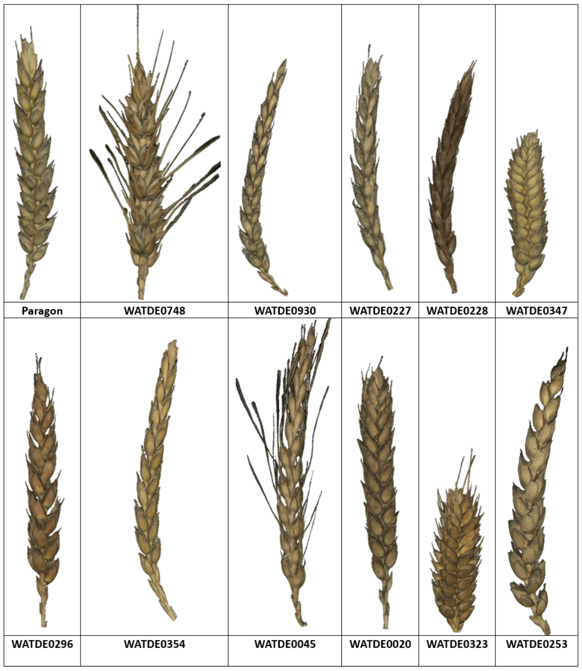

Figure S1: Representative 3D surface-scanning examples demonstrating the diversity of bread wheat shapes.

1.2 Comparaison of volume measurements

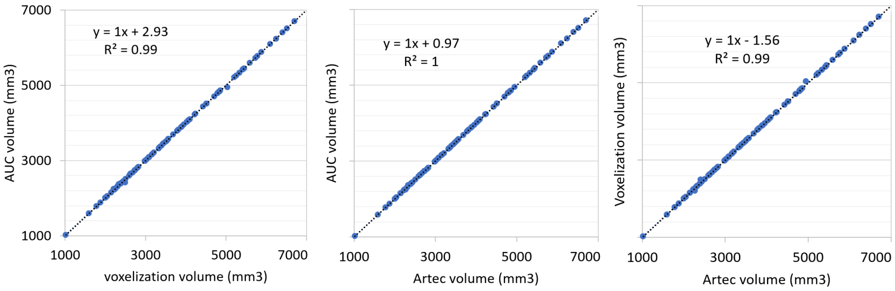

Figure S2: Correlation between the volume of all the spikes using three different methods.

1.3 Cross-sectional area profile of individual wheat spikes

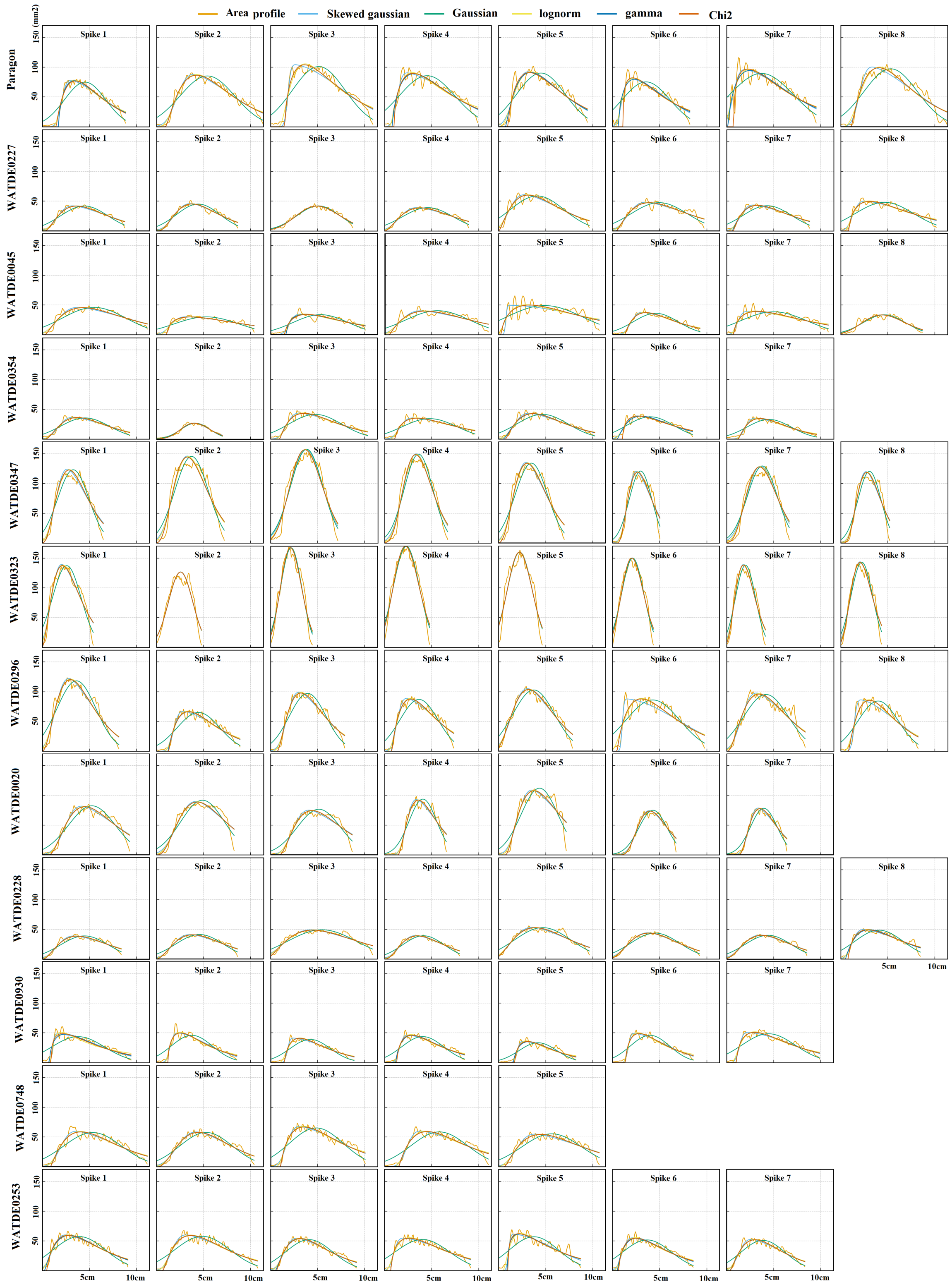

Figure S3: The subplots show individual spike area profile (orange line) alongs spike length overlaid with the following statistical distribution models: skew-Gaussian, lognormal, gamma, and chi-square distribution, see data in Table S15. Each row represent spikes of each genotype, see Tables S10 for model parameters tuning.

1.4 Correlation between TGW and the other traits

The thousand grain weight (TGW) was calculated as the mean of data collected from three plot replicates in each growing season (2022 and 2023), see Table S17. The different genotypes were cultivated in separate fields across the two years. Each plot measured 1 m $\times$ 1 m. The trials were conducted under rainfed conditions, and plants received standard agronomic management, including the application of sulphur (27 kg ha^{-1}) and nitrogen (50 kg ha^{-1}), along with appropriate autumn and spring herbicides, fungicides, and insecticides.

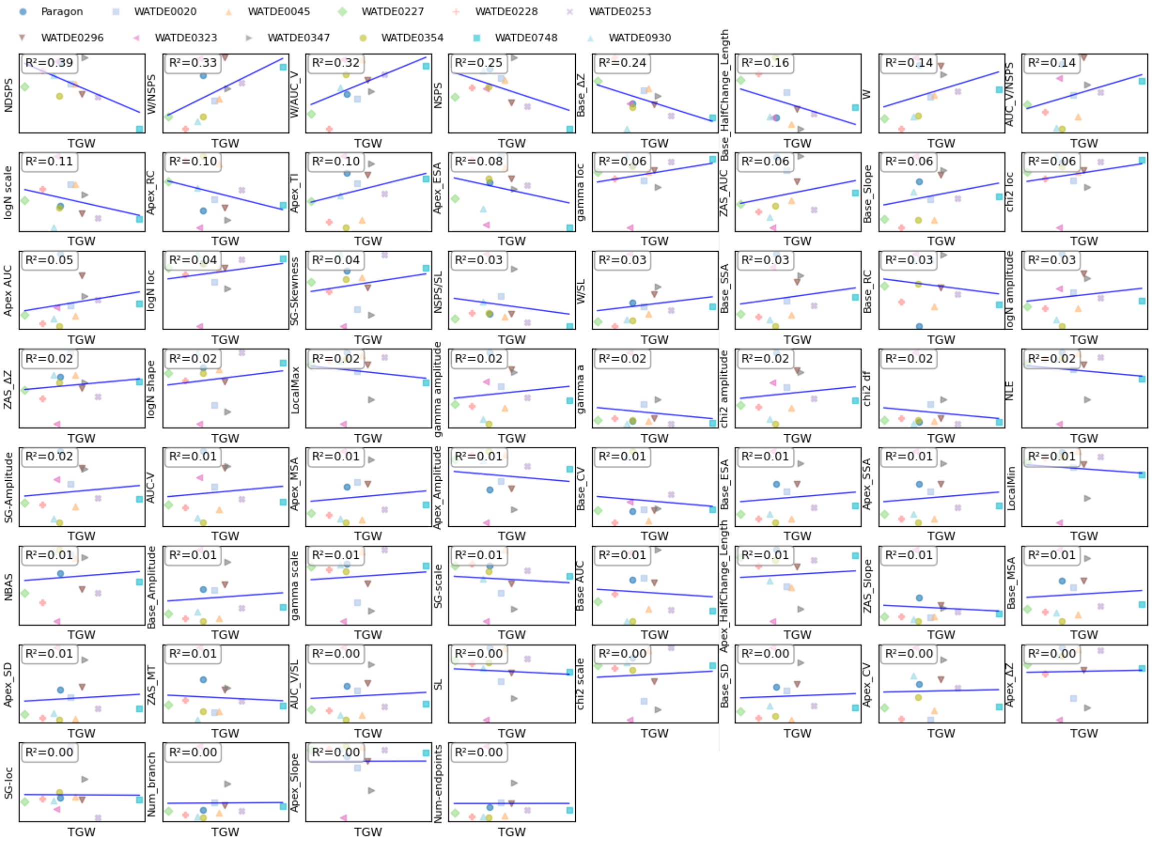

Figure S4: Correlation between thousand grain weight (TGW; x-axis) and spike traits (y-axis). Colored points represent trait values derived from the averaged area profile of each genotype, with R^2 indicating the strength of the relationship.

1.5 Optimal parameters of fitting curves and precision of the fitting

Tables S1, S2, S3, and S4 summarize the fitting results obtained from the averaged area profiles of all spikes in each wheat variety. Correspondingly, Table S10 in the Supplementary Materials lists the optimal parameters of the fitted curves derived from the individual spike area profiles within each variety.

Table S1: Skewed Gaussian optimal parameters for the mean slice area in each variety.

Variety	Amplitude	Loc	Scale	Skewness (α)	MSE	R^2
Paragon	6213.88	15.49	51.58	6.63	31.45	0.95
WATDE0227	3421.94	14.81	51.55	5.53	8.07	0.96
WATDE0228	3334.72	15.29	52.76	4.60	7.34	0.95
WATDE0253	3665.20	11.71	48.98	8.09	8.76	0.97
WATDE0020	4452.62	25.28	34.02	2.90	42.73	0.95
WATDE0296	5308.10	15.06	40.58	4.93	31.69	0.96
WATDE0323	4678.20	13.35	16.36	1.28	136.61	0.93
WATDE0347	5223.66	19.01	24.30	2.32	112.40	0.94
WATDE0354	2353.36	16.49	47.95	5.76	3.13	0.97
WATDE0045	3244.40	15.71	62.14	5.98	7.90	0.93
WATDE0748	3611.00	15.21	45.16	6.86	16.44	0.95
WATDE0930	2557.98	15.01	43.26	8.17	4.34	0.98

Table S2: Log-normal optimal parameters for each variety.

Variety	Amplitude	Shape	Loc	Scale	MSE	R^2
Paragon	6673.46	0.59	-6.93	60.34	37.53	0.95
WATDE0227	3650.20	0.55	-11.14	63.74	7.87	0.96
WATDE0228	3499.15	0.49	-18.15	71.57	6.65	0.95
WATDE0253	4136.61	0.71	-2.55	52.50	12.40	0.95
WATDE0020	4476.24	0.29	-26.07	74.63	39.04	0.95
WATDE0296	5536.89	0.48	-11.27	55.30	34.64	0.96
WATDE0323	4682.80	0.14	-69.78	93.17	130.21	0.93
WATDE0347	5249.22	0.24	-32.15	67.64	101.81	0.95
WATDE0354	2503.05	0.55	-7.48	58.89	3.89	0.96
WATDE0045	3509.34	0.58	-12.27	74.27	8.02	0.93
WATDE0748	3972.01	0.63	-1.95	51.37	19.06	0.94
WATDE0930	2861.27	0.71	2.24	46.06	6.75	0.96

Table S3: Gamma optimal parameters for the mean slice area in each variety.

Variety	Amplitude	Shape (k)	Loc	Scale	MSE	R^2
Paragon	6284.44	2.51	3.59	21.84	33.92	0.95
WATDE0227	3444.76	2.74	0.41	20.76	6.86	0.96
WATDE0228	3340.86	3.11	-3.46	19.81	5.75	0.96
WATDE0253	3806.25	1.88	5.77	26.41	10.27	0.96
WATDE0020	4425.83	6.09	-3.99	9.10	37.24	0.96
WATDE0296	5349.32	3.07	0.99	15.40	29.81	0.96
WATDE0323	4683.00	16.95	-28.51	3.13	127.50	0.94
WATDE0347	5236.72	7.10	-7.38	6.34	95.79	0.95
WATDE0354	2366.18	2.73	3.27	19.22	3.85	0.96
WATDE0045	3267.14	2.61	0.03	25.70	7.13	0.93
WATDE0748	3692.10	2.23	6.81	21.13	17.04	0.95
WATDE0930	2627.43	2.03	8.57	21.55	6.43	0.97

Table S4: Chi-square optimal parameters for the mean slice area in each variety.

Variety	Amplitude	df	Loc	Scale	MSE	R^2
Paragon	6314.49	4.68	5.04	11.56	34.10	0.95
WATDE0227	3444.77	5.48	0.41	10.38	6.86	0.96
WATDE0228	3340.86	6.23	-3.46	9.91	5.75	0.96
WATDE0253	3806.25	3.76	5.77	13.20	10.27	0.96
WATDE0020	4425.84	12.17	-3.99	4.55	37.24	0.96
WATDE0296	5349.32	6.14	0.99	7.70	29.81	0.96
WATDE0323	4683.00	33.89	-28.51	1.57	127.50	0.94
WATDE0347	5236.70	14.21	-7.39	3.17	95.79	0.95
WATDE0354	2366.18	5.47	3.27	9.61	3.85	0.96
WATDE0045	3267.14	5.21	0.03	12.85	7.13	0.93
WATDE0748	3680.80	4.58	6.36	10.31	17.03	0.95
WATDE0930	2627.43	4.07	8.57	10.77	6.43	0.97

1.6 Pearson correlation between all traits

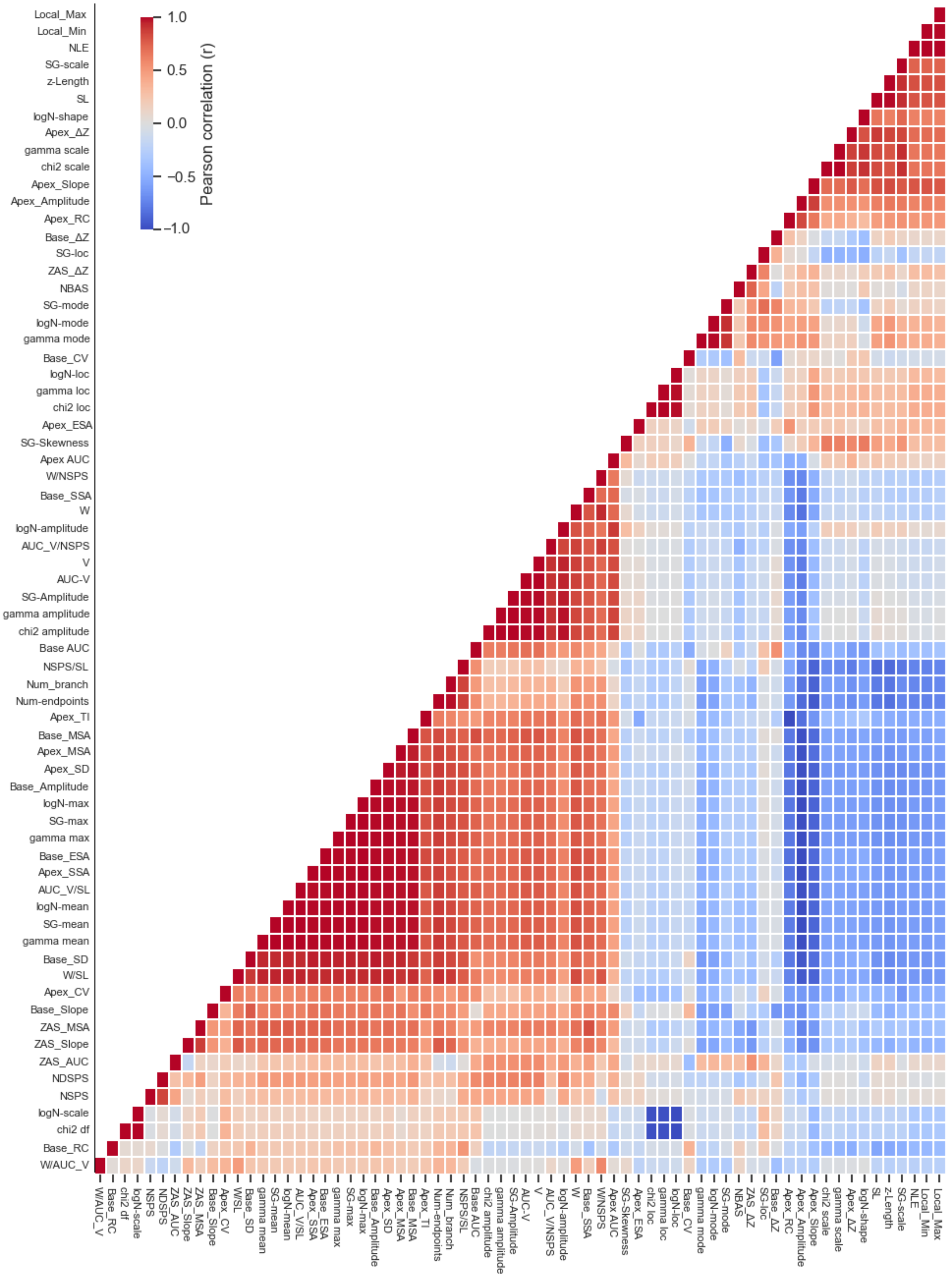

Figure S5: Overview of correlation relationships between traits: pearson's correlation coefficients between the 68 traits. lighter blue and red colors indicates less correlation.

1.7 Abreviation

Table S5: Alphabetical listing of the analyzed traits along with their abbreviations used throughout the study.

**Abbreviation**	**Trait name / Description**
Apex-Δz	Apex length
Apex-Amplitude	Apex area profile amplitude (mm^2)
Apex-AUC	Apex volume (mm^3)
Apex-CV	Apex coefficient of variation (of slice area within segment)
Apex-ESA	Apex end slice area
Apex-MSA	Apex mean slice area
Apex-RC	Apex relative change by the start in area profile
Apex-SD	Apex standard deviation (of slice area within segment)
Apex-Slope	Apex slope
Apex-SA	Apex start slice area
Apex-TI	Apex tapering index
AUC-V	Area under area profile curve (volume)
AUC-V/NSPS	Ratio of area under curve volume by number of spikelets per spike
AUC-V/SL	Ratio of area under curve volume by skeleton length
Artec-V	Spike volume measured by Artec software
Base-Δz	Base length
Base-Amplitude	Base area profile amplitude (mm^2)
Base-AUC	Base volume (mm^3)
Base-CV	Base coefficient of variation (of slice area within segment)
Base-ESA	Base end slice area
Base-MSA	Base mean slice area
Base-RC	Base relative change by the start in area profile
Base-SD	Base standard deviation (of slice area within segment)
Base-Slope	Base slope
Base-SSA	Base start slice area
chi2-amplitude	Chi-square amplitude parameter
chi2-df	Chi-square degree of freedom parameter
chi2-loc	Chi-square location parameter (shift of the distribution)
chi2-scale	Chi-square scale parameter
G-Amplitude	Gaussian amplitude
G-max	Gaussian maximum
G-mean	Gaussian mean
G-mode	Gaussian mode
G-SD	Gaussian standard deviation
Gam-max	Gamma maximum
Gam-mean	Gamma mean
Gam-mode	Gamma mode
gamma-amplitude	Gamma amplitude parameter

Continued on next page

Table S5 (continued)

Abbreviation	Trait name / Description
gamma-loc	Gamma location parameter (shift of the distribution)
gamma-scale	Gamma scale parameter
LogN-amplitude	Log-normal amplitude parameter
LogN-loc	Log-normal location parameter (shift of the distribution)
LogN-max	Log-normal maximum
LogN-mean	Log-normal mean
LogN-mode	Log-normal mode
LogN-scale	Log-normal scale parameter
LogN-shape	Log-normal shape parameter
NBAS	Number of basal aborted spikelets per spike
NCB	Number of connected branches
NDSPS	Number of developed spikelets per spike
NEP	Number of endpoints
NLE	Number of local extremes
NLMax	Number of local maxima
NLMin	Number of local minima
NSPS	Number of spikelets per spike
NSPS/SL	Ratio of number of spikelets per spike by skeleton length
SG-Amplitude	Skewed Gaussian amplitude
SG-loc	Skewed Gaussian location
SG-max	Skewed Gaussian maximum
SG-mean	Skewed Gaussian mean
SG-mode	Skewed Gaussian mode
SG-scale	Skewed Gaussian scale
SG-Skewness	Skewed Gaussian skewness
SL	Skeleton length
TGW	Thousand grain weight
Voxel-V	Spike volume measured by voxelization technique
W	Weight
W/AUC-V	Ratio of weight by area under curve volume
W/NSPS	Ratio of weight by number of spikelets per spike
W/SL	Ratio of weight by skeleton length
ZAS-Δz	Zone of aborted spikelet length (mm)
ZAS-AUC	Zone of aborted spikelet volume (mm^3)
ZAS-MSA	Zone of aborted spikelet Mean slice area (mm^2)
ZAS-Slope	Zone of aborted spikelets slope
z-Length	Projected spike length on z-axis
